# The past, present and future of anti-malarial medicines

**DOI:** 10.1186/s12936-019-2724-z

**Published:** 2019-03-22

**Authors:** Edwin G. Tse, Marat Korsik, Matthew H. Todd

**Affiliations:** 1 School of Chemistry, The University of Sydney, Sydney, NSW 2006 Australia; 2 School of Pharmacy, University College London, London, WC1N 1AX United Kingdom

**Keywords:** Malaria, *Plasmodium*, Mechanism of action, Drug discovery, Drug development

## Abstract

Great progress has been made in recent years to reduce the high level of suffering caused by malaria worldwide. Notably, the use of insecticide-treated mosquito nets for malaria prevention and the use of artemisinin-based combination therapy (ACT) for malaria treatment have made a significant impact. Nevertheless, the development of resistance to the past and present anti-malarial drugs highlights the need for continued research to stay one step ahead. New drugs are needed, particularly those with new mechanisms of action. Here the range of anti-malarial medicines developed over the years are reviewed, beginning with the discovery of quinine in the early 1800s, through to modern day ACT and the recently-approved tafenoquine. A number of new potential anti-malarial drugs currently in development are outlined, along with a description of the hit to lead campaign from which it originated. Finally, promising novel mechanisms of action for these and future anti-malarial medicines are outlined.

**Electronic supplementary material:**

The online version of this article (10.1186/s12936-019-2724-z) contains supplementary material, which is available to authorized users.

## Background

In 2017, the World Health Organization (WHO) estimated that there were 219 million cases of malaria worldwide, an increase of 2 million from the previous year, and as a result there were 435 thousand deaths, or 1190 per day, mostly young children [1]. Encouragingly, since 2000, these figures have decreased by about 37% worldwide, but a number of recent reports have shown that this level is slowly plateauing, emphasizing that there must not be complacency with the current treatment and prevention strategies [2, 3]. There are five species of the *Plasmodium* parasite, with *Plasmodium falciparum* being the most prevalent in Africa and *Plasmodium vivax* being the most prevalent in countries outside Africa. Almost half the world’s population is at risk of contracting malaria, with Africa having the biggest share of cases and deaths of any continent ($\sim$ 90%).

Over the past few years, a number of reviews have been published which evaluate the potential future of anti-malarial drugs. Of note: Triple-anti-malarial drug combinations were examined in 2014 [4]; a review on the numerous strategies currently used in anti-malarial drug discovery was published in early 2017 [5], and an in-depth primer on all aspects of malaria was published in late 2017 [6]. In early 2018, a review was published highlighting the discovery and development of a number of new anti-malarial drug candidates [7, 8].

This review aims to summarize the past, present and future of compounds used to treat malaria. There is a focus on projects supported by the Medicines for Malaria Venture (MMV), a non-governmental organisation that maintains a website [9] highlighting collaborations from the very early stages of drug discovery and lead optimization (e.g. the Open Source Malaria (OSM) project with which the authors are involved [10]), to the progression of lead compounds through clinical trials (e.g. Cipargamin, which has been developed by Novartis [11]) and all the way to the final stages of bringing a drug to market (e.g. Artesunate for injection, developed by Guilin Pharmaceutical). After a brief survey of past medicines, and those that are currently being used, focus will be put on compounds that are currently in development, and in particular the lead optimization campaigns of each. Since it is such a crucial component of modern anti-malarial drug discovery, this review will end with a survey of the most promising mechanisms of action of those compounds in development.

The scope of this review encompasses compounds described by the MMV survey of current anti-malarials, previous reviews on anti-malarial drugs, as well as compounds currently undergoing active clinical trials. The information presented in this review was obtained through structure searching of the relevant compounds in the SciFinder database. Additional references were found through the Web of Science database (Additional file 1).

## Past

**Fig. 1:**
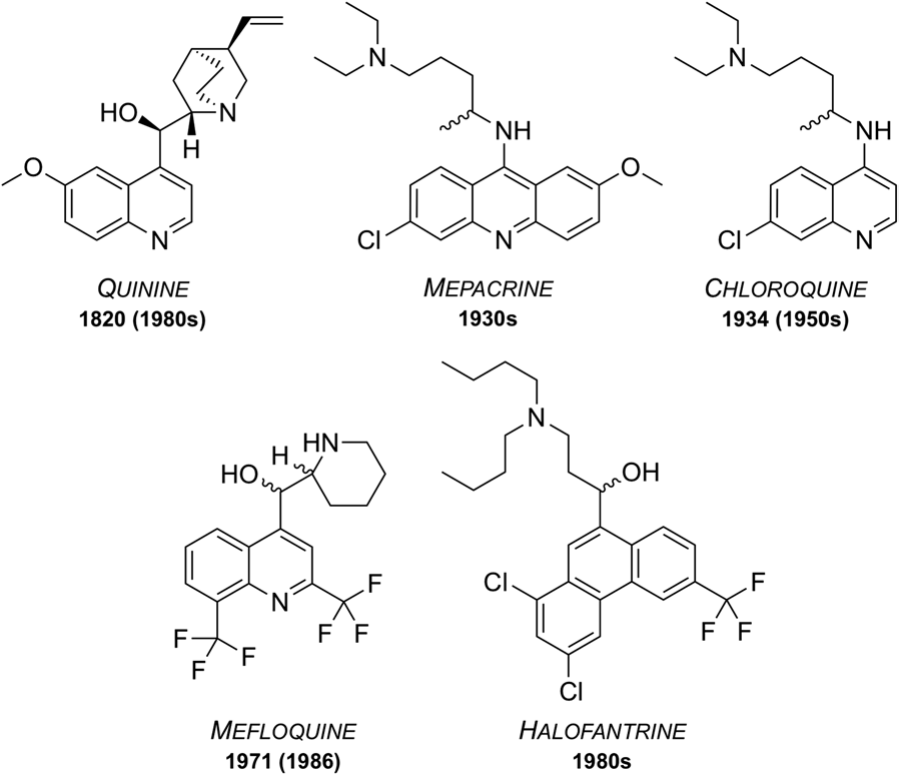
Well known anti-malarial medicines discovered between 1820 and the 1980s. Some are still used today while some have been rendered ineffective due to development of resistant strains or the emergence of undesirable side effects. Dates of first reported resistance are shown in brackets

Since the isolation in 1820 of quinine, the first chemically purified effective treatment for malaria, a number of other natural and synthetic compounds have been developed (Fig. 1). However, as time passed, strains of the parasite began to show signs of resistance towards these drugs, rendering them less effective. Accordingly, their use has ceased or is restricted to particular situations.

### Quinine

First isolated from the bark of the cinchona tree in 1820, quinine has been used as one of the most effective treatments for malaria to date [12]. Resistance was first reported in the 1980s [13] and as of 2006, quinine is no longer used as a front-line treatment for malaria but is still on the WHO’s Model List of Essential Medicines (MLEM) [14] for the treatment of severe malaria in cases where artemisinins are not available.

### Mepacrine

Mepacrine (a.k.a quinacrine) was predominantly used throughout the Second World War as a prophylatic, sold under the trade name Atabrine [15]. This compound is a derivative of methylene blue, another anti-malarial that was discovered in 1891 and found to be an effective treatment for malaria [16, 17]. Its use has declined over the years but methylene blue and its derivatives are, however, the subject of increasing current interest [18] and it is currently in clinical trials as a combination with primaquine (*vide infra*). Mepacrine itself is no longer used today due to the high chance of undesirable side effects such as toxic psychosis [19].

### Chloroquine

During the 1940s, chloroquine (CQ) was used to treat all forms of malaria with few side effects [20]. Resistance to CQ was first reported in the 1950s and over the years many strains of malaria have developed resistance. Indeed, resistant strains (K1, 7GB, W2, Dd2, etc.) of the malaria parasite are now used in potency evaluation assays as a way of showing efficacy [21]. Chloroquine is on the MLEM for the treatment of *P. vivax* in regions where resistance has not developed [14].

### Mefloquine

Mefloquine was developed in the 1970s by the United States Army [22] and is still used today, also being one of the medicines on the MLEM. Originally introduced for the treatment of chloroquine-resistant malaria, it has been used as both a curative and a prophylactic drug. Resistance was first reported in 1986 [23]. It is thought that the structurally related quinoline drugs (such as quinine, mepacrine, chloroquine and mefloquine) act through the disruption of haemoglobin digestion in the blood stage of the parasite [24]. These drugs are commonly used in combination with a complementary drug (e.g. mefloquine and artesunate, sold as Artequin™) to reduce the chance of resistance development to the quinoline family of compounds. Mefloquine is commonly sold in its racemic form under the brand name Lariam®, however it is no longer widely used due to the perception of central nervous system toxicity that has been suggested to affect a large number of its users [25].

### Halofantrine

Developed between the 1960s and 1970s by the Walter Reed Army Institute of Research [26], halofantrine was initially used for treatment against all forms of the *Plasmodium* parasite. Its use has diminished over time due to a number of undesirable side effects, such as the potential for high levels of cardiotoxicity. It is only used as a curative drug and not for prophylaxis due to the high toxicity risks and its unreliable pharmacological properties. Halofantrine is still used today, under the brand name Halfan™, but only in cases where patients are known to be free of heart disease and where infection is due to severe and resistant forms of malaria [27].

## Present

On the WHO Model List of Essential Medicines [14], there are currently listed 14 medicines for curative treatment of malaria and 4 medicines for prophylactic treatment, with the treatments formulated either as single compounds or as combinations. Perhaps the most effective of these are the artemisinin-based combinations which use an artemisinin derivative (short-acting) in combination with one or more complementary compounds (long-acting and possessing different mechanisms of action).

**Fig. 2:**
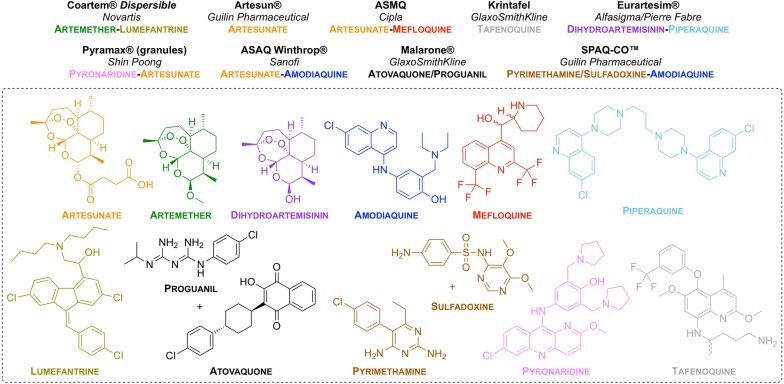
New drug combinations/formulations that have been approved for use. Brand name of the drug (in bold), partnered company (in italics) and drug combination (colour-coded to the structures) are listed

MMV have been involved in progressing nine new anti-malarial treatments based on different formulations/combinations of approved drugs (Fig. 2). All of these compounds are on the MLEM as combination therapies.

### Artemisinin and its derivatives

Artemisinin was first isolated in 1971 by Tu Youyou from the plant *Artemisia annua*, a herb that has commonly been used in Chinese traditional medicine [28]. Due to the great positive impact of artemisinin in combating malaria, Youyou was awarded the joint Nobel Prize in Physiology or Medicine in 2015 for “*her discoveries concerning a novel therapy against malaria*” [29]. Artemisinin has been shown to be efficacious against all multi-drug resistant forms of *P. falciparum*. The most common derivatives of artemisinin are artemether, artesunate and arteether. These semi-synthetic derivatives are prodrugs which are transformed to the active metabolite, dihydroartemisinin. The use of artemisinins has been integral in the fight against malaria with ACT making up the majority of modern day treatments [30]. Although slow to develop, the first report of resistance to artemisinin was in western Cambodia in 2008 [31]. Ten years later, in February of 2018, a report was published identifying more than 30 independent cases of artemisinin resistance in southeast Asia, specifically with resistance to the dihydroartemisinin–piperaquine combination therapy [32].

The mechanism of action (MoA) through which artemisinin acts has been widely debated [33]. The most accepted theory is that the molecule is activated by haem to generate free radicals, which in turn damage proteins required for parasite survival [34, 35]. Still, evidence for a number of other possible mechanisms have been found. In 2013, a computational approach was taken to determine the MoA based around previous studies which identified haem and *Pf*ATP6 (Ca^2+^ transporter) as potential MoAs [36]. More recently in 2015, artemisinin was shown to be associated with the up-regulation of the unfolded protein response (UPR) pathways which may be linked to decreased parasite development [37]. Another study showed that artemisinin was is a potent inhibitor of *P. falciparum* phosphatidylinositol-3-kinase (*Pf*PI3K) [38].

### Amodiaquine

Amodiaquine was first synthesized in 1948 [39]. It is mainly used for the treatment of uncomplicated *P. falciparum* malaria when used in combination with artesunate and is commonly sold under the trade name Camoquine® or Coarsucam™ [40]. Similar to chloroquine, amodiaquine’s MoA is thought to involve complexation with haem and inhibition of haemozoin formation [41].

### Piperaquine

Piperaquine was developed in the 1960s as a part of the Chinese National Malaria Elimination Programme [42]. Initially used throughout China as a replacement for chloroquine, resistance led to its diminished use as a monotherapy. While the MoA of piperaquine is not completely understood, studies have suggested that it acts by accumulating in the digestive vacuole and inhibiting haem detoxification through the binding of haem-containing species [30, 43]. These days, piperaquine is used as a partner drug with dihydroartemisinin (commonly sold under the trade name Eurartesim®).

### Lumefantrine

Lumefantrine (a.k.a. benflumetol) was first synthesized in 1976 as a part of the Chinese anti-malarial research effort “Project 523” which also resulted in the discovery of artemisinin [44]. It is currently sold under the trade name Coartem®. The exact MoA of lumefantrine is unknown, however studies suggest that it inhibits nucleic acid and protein synthesis through the inhibition of $\beta$-haematin formation by complexation with haemin [41]. Lumefantrine is currently used only in combination with artemether.

### Proguanil and atovaquone

Proguanil was first reported in 1945 as one of the first antifolate anti-malarial drugs [45], while atovaquone was first reported in 1991 for the treatment of protozoan infections [46]. The combination of these, commonly sold as Malarone™, has been marketed by GlaxoSmithKline (GSK) since the early 2000s, and has proven to be a very effective anti-malarial due to the synergistic effect of the two components. This is, in large part, due to the different MoAs for each compound. Atovaquone acts as a cytochrome *bc*$_\text {1}$ complex inhibitor which blocks mitochondrial electron transport [47]. Proguanil (when used alone) acts as a dihydrofolate reductase (DHFR) inhibitor through its metabolite, cycloguanil (CG) which disrupts deoxythymidylate synthesis. When used in combination with atovaquone however, proguanil does not act as a DHFR inhibitor but has instead been shown to reduce the concentration of atovaquone required for treatment [48]. Generic atovaquone/proguanil is still available today for the treatment of chloroquine-resistant malaria.

### Pyrimethamine and sulfadoxine

Pyrimethamine (PYR) was developed in the early 1950s by Gertrude Elion and George Hitchings and is now sold under the trade name Daraprim™ [49]. The development of pyrimethamine was a part of the efforts that won Elion, Hitchings and Black the joint Nobel Prize in Physiology or Medicine in 1988 for “*their discoveries of important principles for drug treatment*” [50]. Sulfadoxine was developed in the early 1960s [51]. It is no longer used as a preventative drug due to high levels of resistance. The combination of pyrimethamine and sulfadoxine was approved for use for the treatment of malaria in 1981 and is now commonly sold under the trade name Fansidar®. Both drugs are known to target the parasite folate biosynthesis pathway [52]. Pyrimethamine inhibits dihydrofolate reductase, while sulfadoxine inhibits dihydropteroate synthetase.

### Pyronaridine

Pyronaridine was first synthesized in the 1970s at the Institute of Chinese Parasitic Disease [53, 54]. It has been found to be efficacious against chloroquine resistant strains and has been in use for over 40 years, sold under the trade name Pyramax® (in combination with artesunate). Like lumefantrine, pyronaridine has been found to act through the inhibition of $\beta$-haematin formation [55].

### Tafenoquine

First discovered in 1978 at the Walter Reed Army Institute of Research, Tafenoquine (TQ) was recently approved by the United States Food and Drug Administration for use as the first new single-dose for the radical treatment of *P. vivax* malaria in over 60 years [56]. TQ is thought to be a prodrug which is metabolized to the active quinone TQ, however the MoA is not well known [57]. It is currently sold under the brand name Krintafel.

## Future

**Fig. 3:**
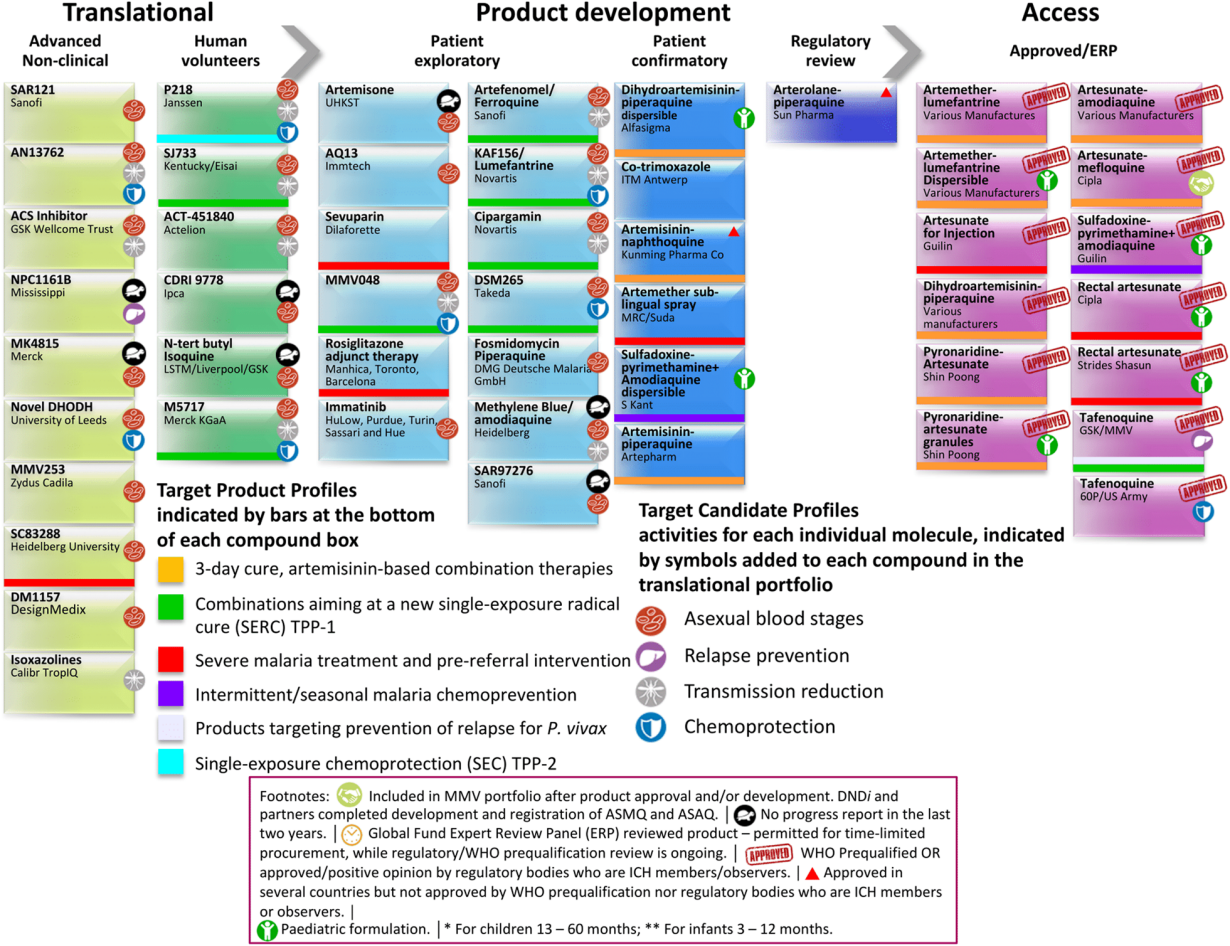
Snapshot of the projects supported by MMV at different stages of the drug discovery and development pipeline (Adapted from the MMV-supported projects webpage [58])

Since its establishment in 1999, the Medicines for Malaria Venture has been frontlining the discovery and development of new medicines for the treatment of malaria. The potential for these compounds to act as new anti-malarials is judged by a number of requirements: novel modes of action with no cross-resistance to current drugs; single-dose cures (artesunate and chloroquine are unable to do this); activity against both the asexual blood stages that cause disease and the gametocytes responsible for transmission; compounds that prevent infection (chemoprotective agents); and compounds that clear *P. vivax* hypnozoites from the liver (anti-relapse agents) [59, 60]. MMV has been aiding the fight against malaria by partnering with universities and pharmaceutical companies around the world to bring new anti-malarials to market (Fig. 3).

**Fig. 4:**
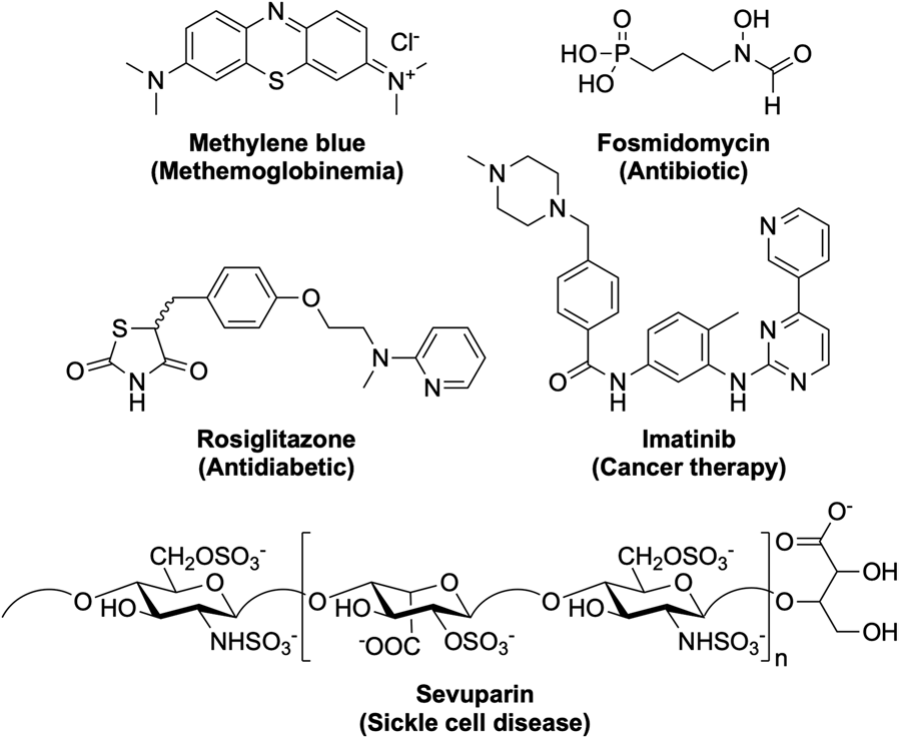
New anti-malarial compounds in development as a result of drug repurposing. Original indications for the drugs are shown in parentheses. Many of these repurposed drugs are already in Phase II trials as new potential anti-malarials

Besides the traditional drug discovery and development methods for the identification of new anti-malarials that will be described below, there are a number of other ways in which a new anti-malarial drug may be discovered. One way, as previously mentioned, is through the exploration of new combinations and formulations of current anti-malarial drugs. This may help overcome issues with resistance to a particular component or may assist in the delivery of the drug allowing it to be more effective. Alternatively, existing drugs used for other purposes may be found efficacious against malaria and subsequently be repurposed as a new anti-malarial treatment. This can be advantageous since these compounds may have already shown good biological properties and may also reveal novel MoAs. Examples include those shown in Fig. 4.

**Methylene blue**, a drug for the treatment of methaemoglobinemia. Last completed Phase II trials in 2017 (NCT02851108) as a combination with primaquine. **Fosmidomycin**, an antibiotic. Most recently in Phase II trials in 2015 (NCT02198807) as a combination with piperaquine. **Rosiglitazone**, an antidiabetic drug. Currently in clinical trials as an adjunctive therapy for severe malaria (NCT02694874). **Imatinib**, a cancer therapy drug. Currently in Phase II trials (NCT03697668) as a triple combination with dihydroartemisinin-piperaquine. **Sevuparin**, a drug for the treatment of sickle cell disease. Last in Phase I/II trials in 2014 (NCT01442168) as a combination with atovaquone-proguanil.

**Fig. 5:**
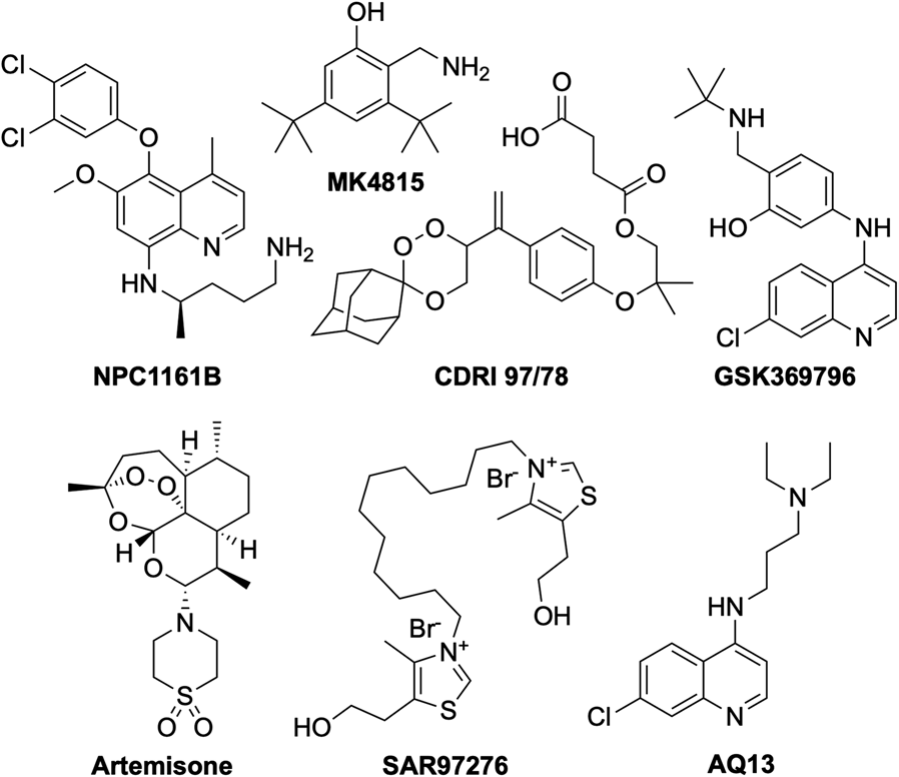
New anti-malarial drug candidates that have reported no significant progress in the last 2 years. These compounds may have encountered issues during preclinical or early clinical studies

The following seven compounds were discovered and developed with the hope of progressing into clinical trials as potential new anti-malarial candidates (Fig. 5). However, over the past two years, progress in the development of these compounds has slowed, making the fate of these drug candidates less clear.

The chiral 8-aminoquinoline derivative, **NPC1161B** was developed at the University of Mississippi and was still in preclinical studies in 2014 [61–64]. **MK4815** was developed in 2012 at Merck but is still in preclinical studies due to safety issues [65]. **CDRI 97/78** is a fast-acting novel trioxane anti-malarial first synthesized in 2001 by a team at the Council of Scientific and Industrial Research in India [66]. Having passed all preclinical studies, it was last seen to have completed first-in-human Phase I trials in 2014 [67]. Developed at the Liverpool School of Tropical Medicine in 2009, ***N-tert butyl isoquine/GSK369796*** was designed as an alternative to amodiaquine [68, 69]. It completed preclinical studies [70], and was last in Phase I trials in 2008 (NCT00675064). **Artemisone** is a second-generation semi-synthetic artemisinin derivative developed at the Hong Kong University of Science and Technology, that has previously been shown to be as efficacious as artesunate, with minimal neuro- and cytotoxicity and a comparably low cost of production [71]. It was withdrawn from Phase II/III trials in 2010 (NCT00936767). The bisthiazolium salt, **SAR97276**, was discovered and developed by Sanofi in 2005 [72], however further investigation was terminated in 2012 after Phase II trials (NCT01445938). **AQ-13** is a chloroquine derivative that was first described in 1946 [73]. While only differing to CQ in the amine side-chain, this difference has been linked to its increased efficacy against CQ-resistant strains [74]. It has a MoA and pharmacokinetic properties similar to that of CQ [75, 76]. **AQ-13** last completed Phase II trial at the end of 2017 (NCT01614964) [77], however there has been no mention of any following active trials so the ongoing status of this compound is unclear.

The compounds in the remainder of this section are currently still actively being pursued. For ease of visualisation throughout in this section, a graphical summary of each compound structure and its code will be displayed, along with key physical and biological properties identified during the hit to lead campaigns (Additional file 2).

### Preclinical

Once a lead compound has been identified, optimization of the structure can begin. This largely involves investigation into the structure activity relationship (SAR) of the drug, optimising for properties such as potency (both in vitro and in vivo), solubility and metabolic stability. The candidate must also be assessed for any possible toxicity (e.g. dosing, cytotoxicity/genotoxicity levels, etc.).

**Fig. 6:**
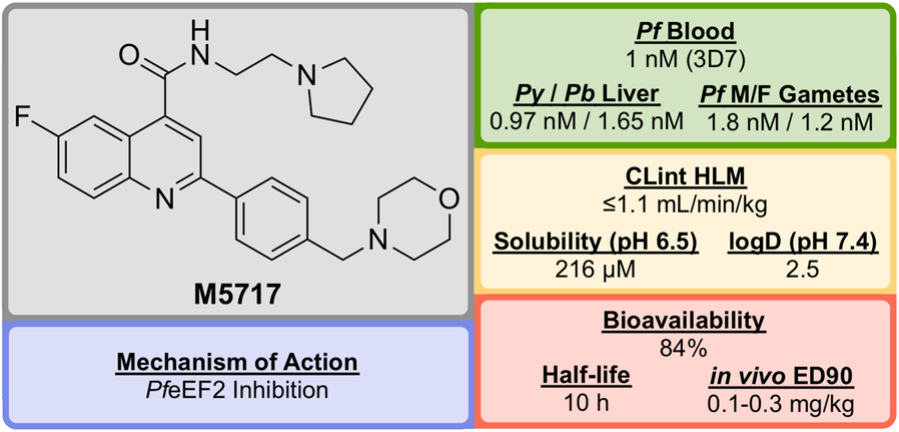
Key biological and physical properties of **M5717**

**M5717** (Fig. 6) was developed in 2015 by a team led by the Drug Discovery Unit (DDU) in Dundee and was shown to have potent activity against multiple stages of the *Plasmodium* parasite *via* a novel mechanism of action [78, 79].

**Fig. 7:**
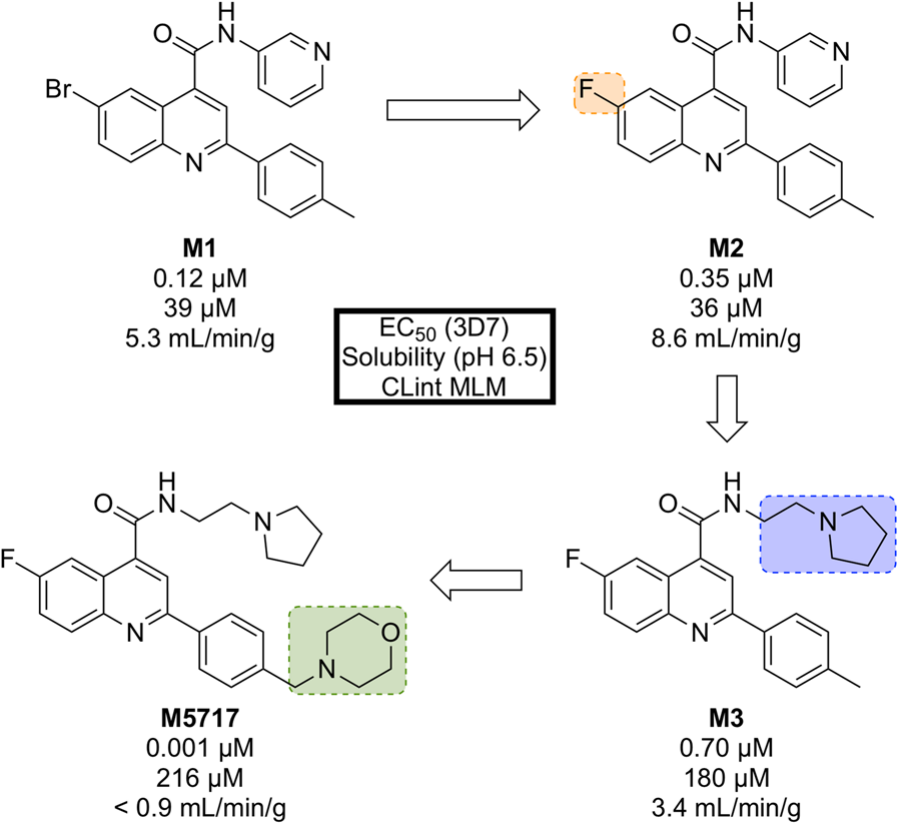
Key stages in the hit to lead pathway of **M5717**. Initial replacement of Br for F, replacement of pyridine with ethylpyrrolidine, and addition of a morpholine fragment led to the optimized compound **M5717**

Initial phenotypic screening of the Dundee protein kinase scaffold library against the 3D7 multi-drug-resistant *P. falciparum* strain identified a compound (**M1**, Fig. 7) that possessed high potency against the parasite, albeit with poor physicochemical properties. Optimization of this structure (via **M2** and **M3**) led to improvements across the board (**M5717**).

In addition to the nanomolar activity against the 3D7 strain, **M5717** has shown almost equal potency against a number of other drug-resistant strains (K1, W2, 7G8, TM89C2A, D6 and V1/S) as well as similar potencies across multiple life cycle stages (liver schizonts, gametocytes and ookinetes).

**M5717** was found to be as effective as current anti-malarial drugs (chloroquine, mefloquine, artemether, dihydroartemisinin and artesunate) when evaluated in the in vivo *Plasmodium berghei* mouse model for single-dose efficacy showing > 99% reduction in parasitaemia at doses of 4 $\times$ 30 mg/kg *p.o. q.d.* and an ED$_{90}$ of 0.1–0.3 mg/kg.

Due to its novel MoA (*Pf*eEF2 inhibition, *vide infra*) and its ability to clear blood-stage parasites completely, **M5717** satisfies the requirements to be a long duration partner and could be used as part of a combination therapy with a fast-acting compound [80]. Additionally, the compound has shown the ability to act as a transmission-blocking drug (stage IV–V) and also to be effective for chemoprotection.

In late 2017, **M5717** was cleared for progression from development to Phase I clinical trials for volunteers in Australia (NCT03261401).

**Fig. 8:**
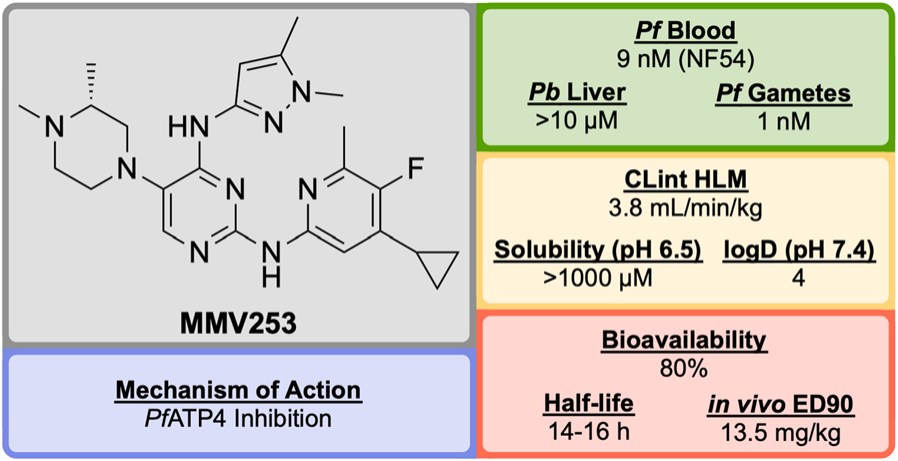
Key biological and physical properties of **MMV253**. logD and in vivo ED_90_ kindly provided by V. Sambandamurthy, S. Hameed P. and S. Kavanagh, personal communication, 2018

Identified by AstraZeneca in 2015, **MMV253** (Fig. 8) is a novel triaminopyrimidine (TAP) that has shown good in vitro potency and in vivo efficacy, and acts through another novel MoA [81].

**Fig. 9:**
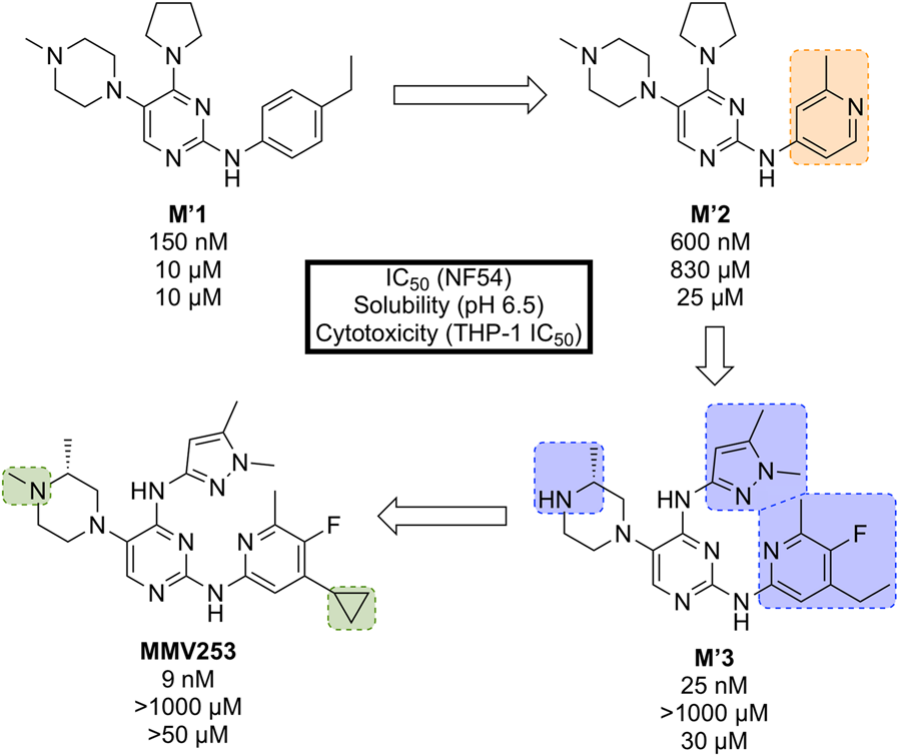
Key stages in the hit to lead pathway of **MMV253**. Initial replacement of ethylbenzene on **M’1** with 2-methylpyridine resulted in lower hERG affinity and improved solubility. Substitution of the pyrrolidine in **M’2** with an imidazole containing an amine spacer further improved solubility and greatly improved the potency. Addition of an *N*-methyl group and a cyclopropane moiety led to the optimized compound **MMV253**

High-throughput screening of 500,000 compounds from AstraZeneca’s library against blood stage *P. falciparum* resulted in the identification of a promising series of TAPs. The initial hit (**M’1**, Fig. 9) suffered from hERG inhibition and poor solubility which, through lead optimization, was improved upon to give a compound that possessed high potency and desirable pharmacokinetic properties (**MMV253**).

When screened against numerous mutant resistant strains with various mechanisms of resistance, **MMV253** showed no spontaneous reduction in potency which can be attributed to its novel MoA (*Pf*ATP4 inhibition, *vide infra*). Good in vitro-in vivo correlation (IVIVC) was shown with a predicted human half-life of $\sim$ 36 h (which is long compared to another fast-killing drug, artemisinin, which has a human half-life of 1 hour).

As of late 2016, the pharmaceutical company Cadila Healthcare owns the license for the compound series and is now doing further lead development in order to progress the drug through preclinical trials [82].

**Fig. 10:**
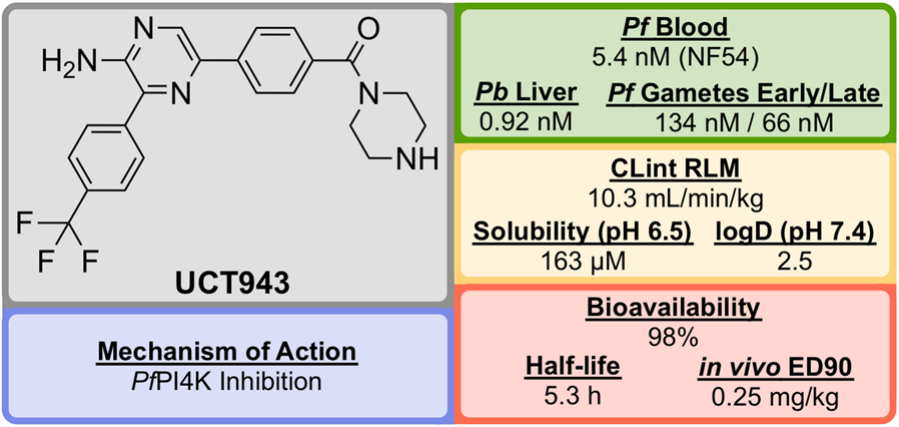
Key biological and physical properties of **UCT943**

**UCT943** (Fig. 10) was identified in 2016 by a team at the University of Cape Town, South Africa and is a key compound in a novel class of 2-aminopyrazine anti-malarials that has shown single dose curing ability in vivo and potential as a clinical candidate [83].

**Fig. 11:**
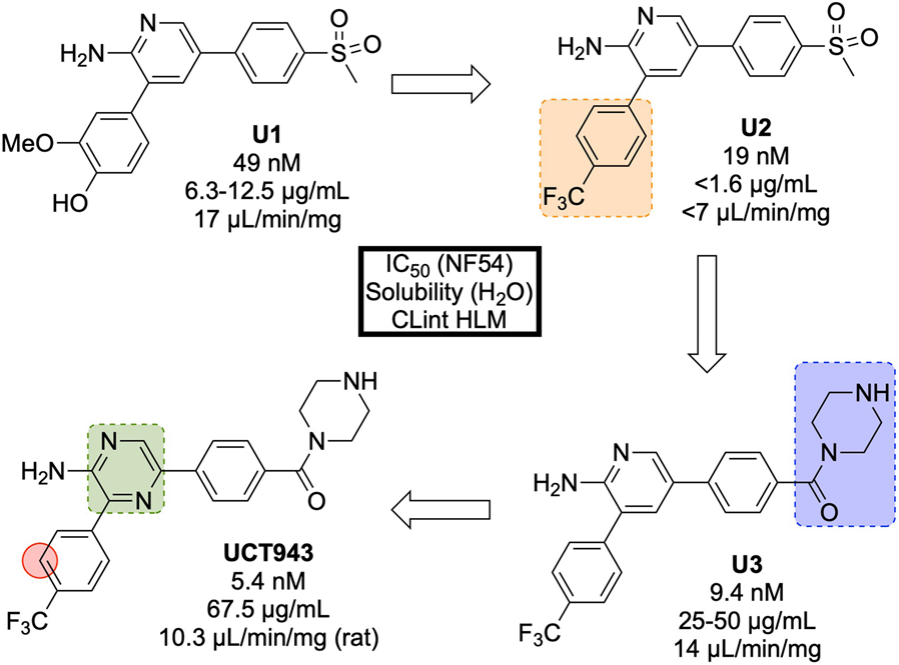
Key stages in the hit to lead pathway of **UCT943**. Initial change the phenyl substituents with a single trifluoromethyl group led to greater in vivo stability. Introduction of piperazine amide instead of methylsulfonyl and a pyrazine instead of a pyridine led to the improved solubility and potency of the optimized compound. Surprisingly, introduction of a nitrogen in the red circle resulted in complete inactivity in vivo

The original 3,5-diaryl-2-aminopyridine series was identified from a high-throughput screen of 36608 compounds from the commercially available SoftFocus kinase library [84]. The initial hit (**U1**, Fig. 11) showed promising in vitro activity against the drug-sensitive NF54 strain (IC$_{50}$ = 49 nM) but suffered from poor solubility and high metabolic clearance. To address the poor measured in vivo stability, the labile hydroxy and methoxy groups were replaced by a single trifluoromethyl group (**U2**), but this change resulted in a significant loss of solubility. Significant improvements in solubility and potency were obtained by first replacing the mesyl group with a piperazine carboxamide group (**U3**) and subsequently introducing another nitrogen atom into the pyridine ring (**UCT943**) [85, 86].

In the *P. berghei* mouse model, **UCT943** has shown the ability to cure malaria at doses of 4 $\times$ 10 mg/kg and has an ED$_{90}$ of 1 mg/kg. Interestingly, another closely related molecule (with a nitrogen instead of a carbon in the red circle) which was evaluated during the SAR study, displayed similar in vitro anti-malarial activity (IC$_{50}$ = 9.1 nM) and solubility (198 $\mu$M) but showed a complete lack of in vivo activity with a < 40% reduction in parasitaemia using a comparable dosage.

**UCT943** is potent across multiple parasite life stages of both *P. falciparum* and *P. vivax*. Its target is *Plasmodium* phosphatidylinositol-4-OH kinase (*Pf*PI4K), which has also been implicated as the target for MMV048 (*vide infra*) [87]. **UCT943** was in originally in place as a back-up to MMV048, however, due to preclinical toxicity, this candidate has been withdrawn.

**Fig. 12:**
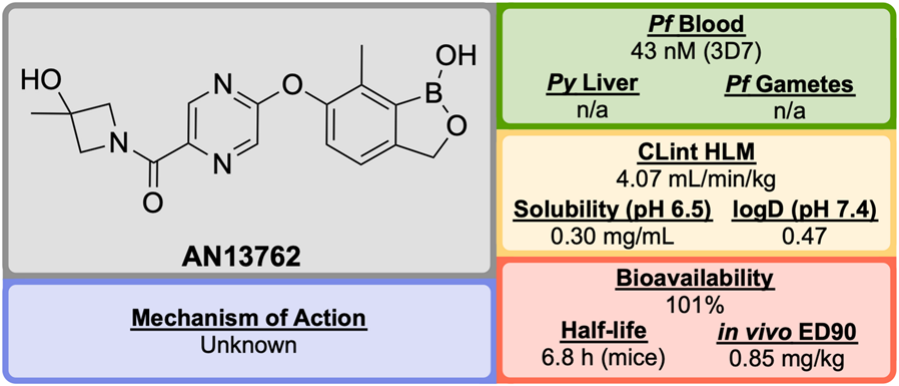
Key biological and physical properties of **AN13762**. Solubility kindly provided by Y.-K. Zhang, personal communication, 2018

From a discovery process by Anacor Pharmaceuticals that began in 2010 with a novel class of benzoxaborole anti-malarial compounds [88], **AN13762** (Fig. 12) emerged in 2017 as the lead compound, showing excellent activity in vitro and in vivo, multi-strain efficacy and the ability to perform as a rapid-acting drug [89].

**Fig. 13:**
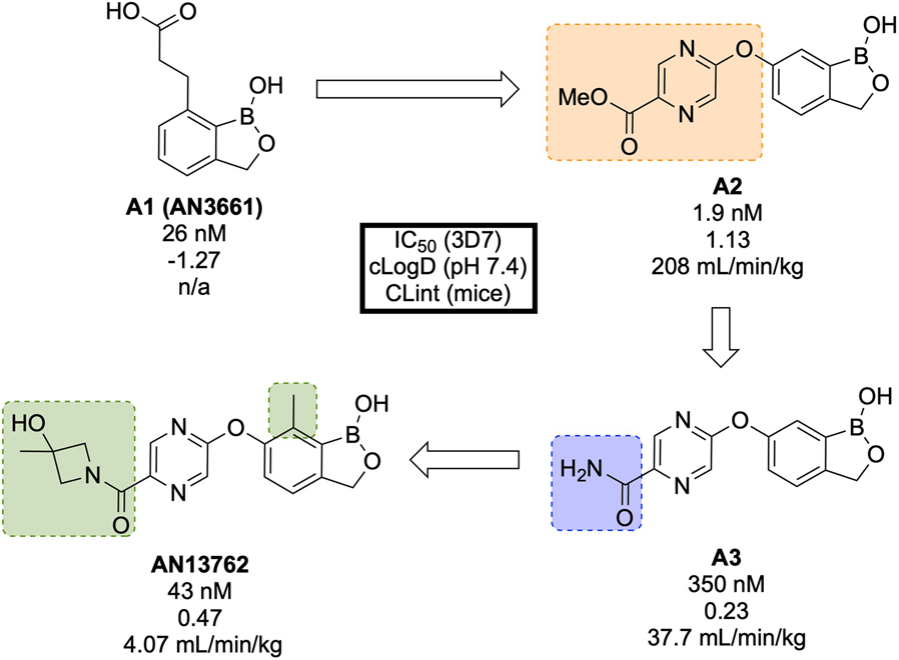
Key stages in the hit to lead pathway of **AN13762**. Initial replacement of the carboxylic acid chain with a pyrazine, and subsequent switch of the ester to a substituted amide helped to improve in vivo stability and bioavailability leading to the optimized compound

By screening a library of boron-containing compounds (previously shown to have selective activity against fungi, bacteria, parasites and inflammation) in a whole cell assay against *P. falciparum*, the initial hit compound, **AN3661** (**A1**, Fig. 13), was identified to have potent in vitro activity against the 3D7 strain (IC$_{50}$ = 26 nM). Further SAR and optimization studies led to the development of **A2** in which the alkylcarboxylic acid chain was moved and replaced by a substituted pyrazine ether. This compound showed greater potency but still suffered from high metabolic clearance [90]. Replacement of the ester group with an amide group (**A3**) led to improved metabolic stability and bioavailability, with a significant decrease in potency. Modification of the primary amide to a cyclic tertiary amide, and introduction of a methyl group on the benzoxaborole gave the lead compound **AN13762** which possessed improved potency and metabolic stability [89].

**AN13762** has been shown to be equally potent across a wide range of drug resistant strains. The drug has displayed similar in vivo clearance rates when compared to artesunate. There is no inherent genotoxicity (as shown in Ames assays and in vivo rat micronucleus studies), and no cytotoxicity at concentrations up to 100 μM in human cell lines [89].

The precise mechanism of action for **AN13762** remains unknown, though initial MoA studies on hit compound **AN3661** identified a potential target as the *P. falciparum* cleavage and polyadenylation specificity factor (*Pf*CPSF3) [91]. **AN13762** has proceeded into the preclinical phase, with first-in-human studies planned for 2019 [58].

**Fig. 14:**
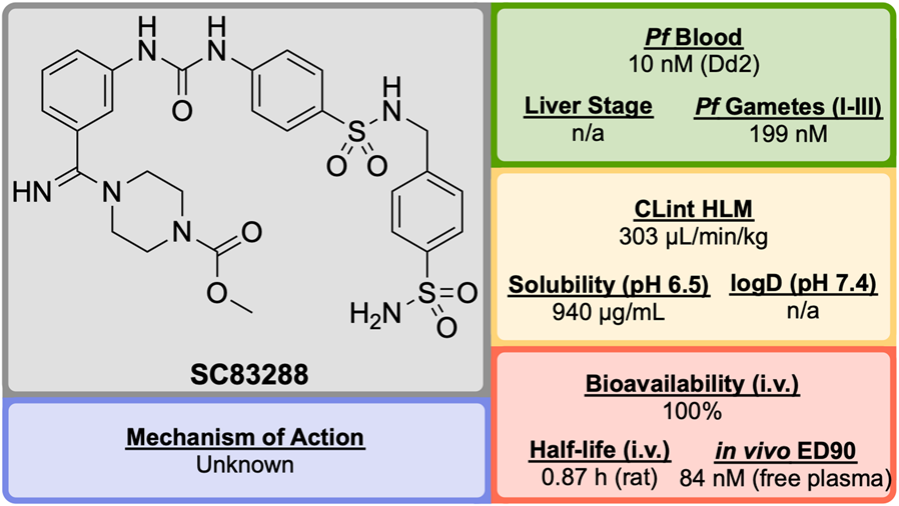
Key biological and physical properties of **SC83288**. In vivo ED_90_ kindly provided by M. Lanzer, personal communication, 2018

Developed in 2017 by a team at Heidelberg University, **SC83288** (Fig. 14) is an amicarbalide derivative that possesses a novel chemotype for current anti-malarials, may have a potentially new MoA and has shown the ability to be a fast-acting drug for the intravenous treatment of severe malaria [92].

**Fig. 15:**
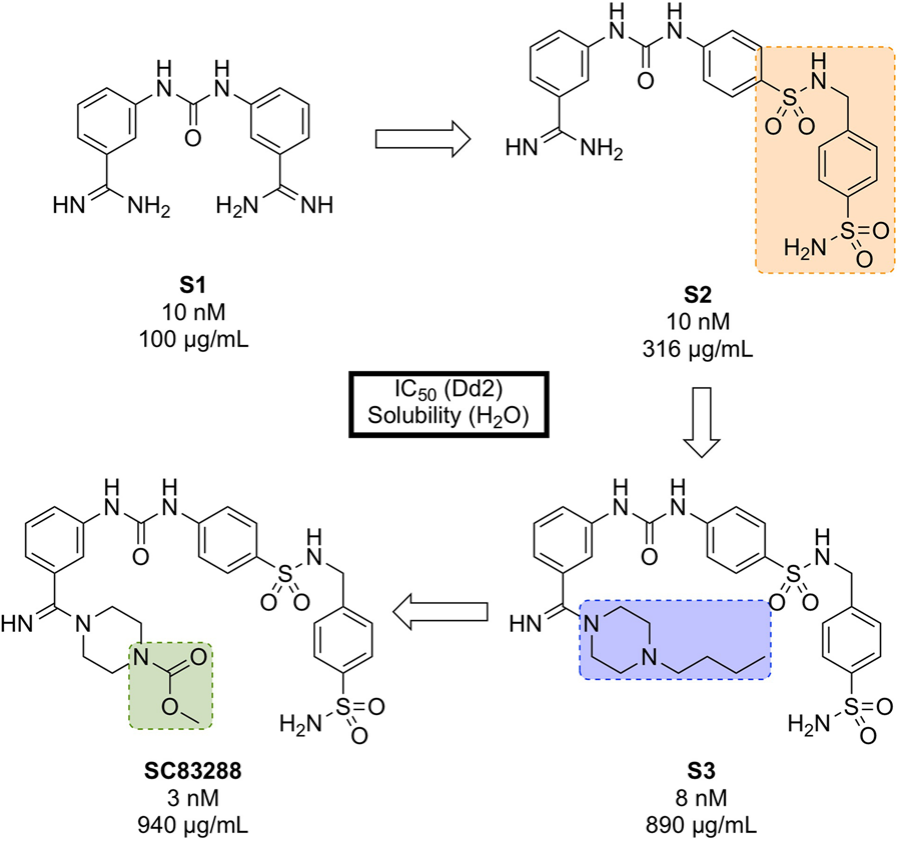
Key compounds in the discovery of **SC83288**. Initial modification of one amidino group with a sulfonamide linker (**S2**) resulted in improved solubility. Further modification of the remaining amidine group with substituted piperazine moieties ultimately led to the optimized compound with good solubility, permeability and high potency

An *in silico* screen of a library of small molecule compounds for their ability to dock into *P. falciparum* lactate dehydrogenase led to the identification of amicarbalide (**S1**, Fig. 15), which was found to be highly potent (IC$_{50}$ = 10 nM) against the Dd2 strain [93]. In order to overcome a potential DNA binding effect, an amidine group was replaced with a sulfonamide linker leading to **S2** which possessed better solubility and improved metabolic stability. Further modification of the other amidine group with a substituted piperazine ring (**S3**) led to improved potency and water solubility, but the compound suffered from poor permeability. Ultimately, replacement of the butyl chain with an acetyl group led to the highly potent lead compound **SC83288**.

**SC83288** has been shown to be potent against a number of drug-resistant strains at IC$_{50}$ values of < 20 nM and is also efficacious against early stage (I–III) gametocytes (IC$_{50}$ = 199 nM) but not against late stage gametocytes (IV and V). It has an excellent safety profile, with no cytotoxicity, genotoxicity or hERG binding. In the *Plasmodium vinckei* rodent malaria model, **SC83288** was able to fully cure the infection at a dose of 4 $\times$ 20 mg/kg i.p. *q.d.* with no resurgence of infection. It is however, completely inactive against *P. berghei*.

While the exact MoA of SC83288 is unknown, the generation of resistant clones has identified *Pf*ATP6 as a possibly relevant target. However, it has been shown that SC83288 does not directly inhibit this target suggesting *Pf*ATP6 may have a less direct role in the mechanism of **SC83288**. *Pf*MDR2 has been identified as another possible mechanism of resistance, facilitating the clearance of the drug from the parasite. **SC83288** has been evaluated against artemisinins, showing no cross resistance and highlighting its potential as an alternative to artesunate for the treatment of severe malaria when combined with a slow-acting partner drug [94].

**Fig. 16:**
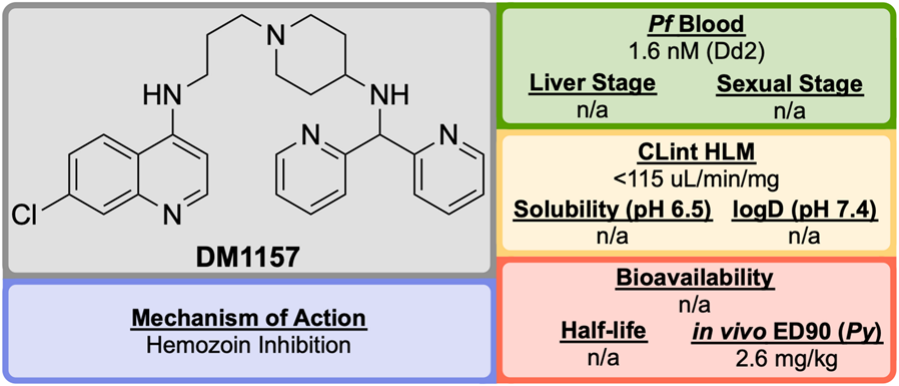
Key biological and physical properties of **DM1157**. CLint HLM and in vivo ED_90_ kindly provided by D. Peyton, personal communication, 2018

Discovered in 2010 by a team at Portland State University and further developed by DesignMedix, **DM1157** (Fig. 16) is part of a class of compounds known as “reversed chloroquines” (RCQs), designed to overcome chloroquine-resistant and chloroquine-sensitive strains of the malaria parasite. The compound has been shown to be efficacious in vitro and in vivo [95].

**Fig. 17:**
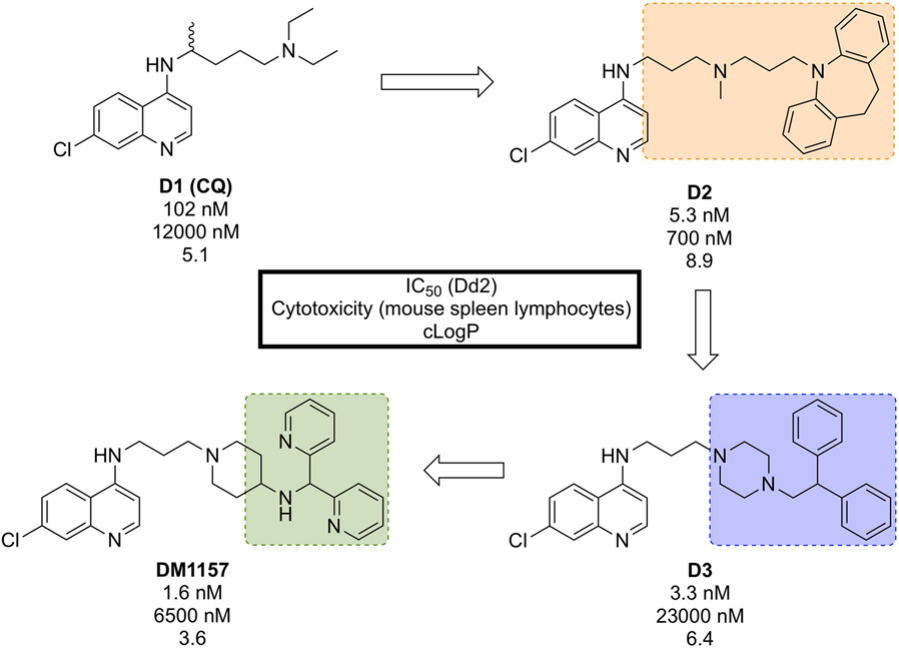
Key compounds in the discovery of **DM1157**. Initial combination of the reversal agent, imipramine, with the CQ core resulted in the potent RCQ compound **D2**. Subsequent replacement of the reversal agent with 1-(2,2-diphenylethyl)piperazine (**D3**), and further modification with pyridine rings led to improved potency and cLogP values for the optimized compound

CQ resistance is known to be linked to the *P. falciparum* chloroquine resistance transporter (*Pf*CRT): mutations to this target facilitate the expulsion of CQ from the parasite. A class of molecules has been identified, so-called “reversal agents”, that can inhibit *Pf*CRT, thus slowing the exportation of CQ from the parasite. By combining the chloroquinoline core of CQ (**D1**, Fig. 17) with the known reversal agent, imipramine, the first RCQ (**D2**) was designed, but this molecule suffered from poor bioavailability and metabolic stability [96]. Subsequent SAR studies resulted in the substitution of the imipramine motif with a 1-(2,2-diphenylethyl)piperazine moiety which led to a compound (**D3**) that was more stable to metabolic cleavage, but suffered from a high cLogP. In order to overcome this, the two phenyl groups were replaced with pyridines and the piperazine replaced with an aminopiperidine resulting in the lead compound, **DM1157** that possessed a lower cLogP value (3.6) while still maintaining high potency against both CQ-resistant (Dd2 = 1.6 nM) and CQ sensitive strains (D6 = 0.9 nM) when compared to CQ (cLogP = 5.1, Dd2 = 102 nM, D6 = 6.9 nM).

In a *P. berghei* rodent model, **DM1157** showed good efficacy both orally and subcutaneously. Most notably, a > 99.9% reduction in parasitaemia was seen at an oral dose of 4 $\times$ 30 mg/kg with 2/3 mice cured 30 days post infection. **DM1157** has also been evaluated against *P. falciparum* and *P. vivax* multi-drug resistant field isolates in Indonesia and was found to be threefold more potent than CQ in both species [97].

CQ is known to bind to heme and inhibit $\beta$-haemozoin formation. **DM1157** (and other RCQ compounds) have been shown also to act in this manner, but with much higher levels of inhibition of $\beta$-haemozoin both in vitro and in vivo. **DM1157** is currently in Phase I trials to evaluate its safety and pharmacokinetics in humans (NCT03490162).

### Translational (human volunteers)

Upon the completion of preclinical trials, the drug will have either passed or failed the required safety standards and pharmacokinetic profiling. For a compound that has passed these requirements, trials may then be conducted in human volunteers in order to show its efficacy as a potential treatment.

**Fig. 18:**
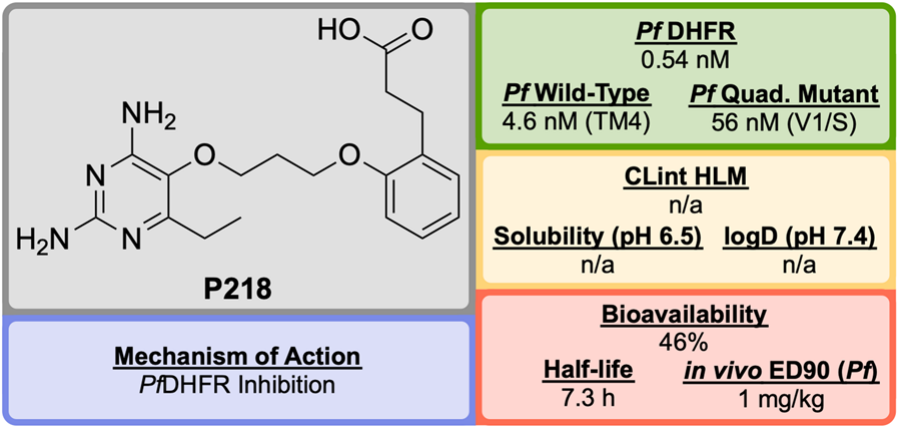
Key biological and physical properties of **P218**

**P218** (Fig. 18), discovered by BIOTEC Thailand in 2012, is an antifolate anti-malarial drug bearing resemblance to the 2,4-diaminopyrimidine core structure of PYR, and is highly selective for the *P. falciparum* dihydrofolate reductase (*Pf*DHFR) [98].

**Fig. 19:**
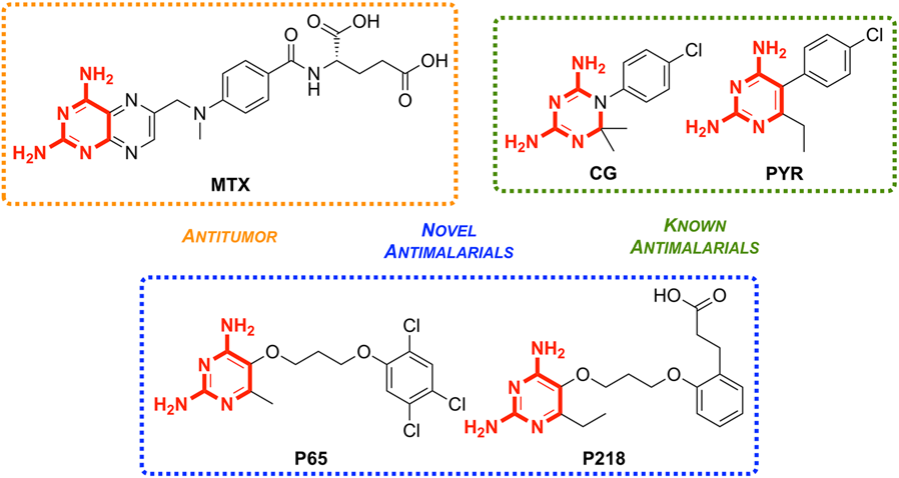
Key compounds in the discovery of **P218**. The key 2,4-diaminopyrimidine core highlighted in red can be found in a number of DHFR inhibitors

Unlike the typical high-throughput screening that is used for the identification of hit compounds in medicinal chemistry, **P218** was identified through careful examination of the cocrystal structures of known *Pf*DHFR inhibitors and their substrates. The initial observation that 2,4-diaminopyrimidines acted as antagonists to folic acid led to the discovery and development of methotrexate (**MTX**) as an antitumor drug (Fig. 19). By examining the binding interactions with DHFR, the structure of **P65** was identified. A total of over 200 compounds (not described in the original report) were designed and synthesized after further examination of potential interactions with amino acid residues, with the optimized structure of **P218** being identified as the best compound.

The 2,4-diaminopyrimidine scaffold of **P218** has been found to bind deep in the active site of *Pf*DHFR in both wild-type and mutant strains. This, along with the hydrogen bonding interaction of the carboxylate group with an Arg residue at the opposite end of the active site results in tighter binding and a longer residence time when compared to **PYR**. Since **P218** is contained almost entirely within the dihydrofolate binding site, the strength of the binding should be strong enough to overcome any amino acid mutations, thus minimising the chance of drug-resistant mutations to arise. The novel two-step mechanism of action for binding to *Pf*DHFR (*vide infra*) allows **P218** to overcome resistance that has emerged from the use of pyrimethamine. P218 has also shown high selectivity to the binding of malarial over human DHFR, which translates into reduced toxicity.

In vivo studies have shown **P218** to be highly efficacious against *P. falciparum* and *Plasmodium chabaudi* in mice with ED$_{90}$ values of 1 mg/kg and 0.75 mg/kg respectively. In the in vitro and in vivo potency assays that were run, **P218** was found to be more potent than PYR in all cases.

Along with its high potency and good safety profile, **P218** has the potential to be a replacement for **PYR** combination with **CG** in areas where *Pf*DHFR resistance has emerged. P218 has currently completed Phase I trials (NCT02885506).

**Fig. 20:**
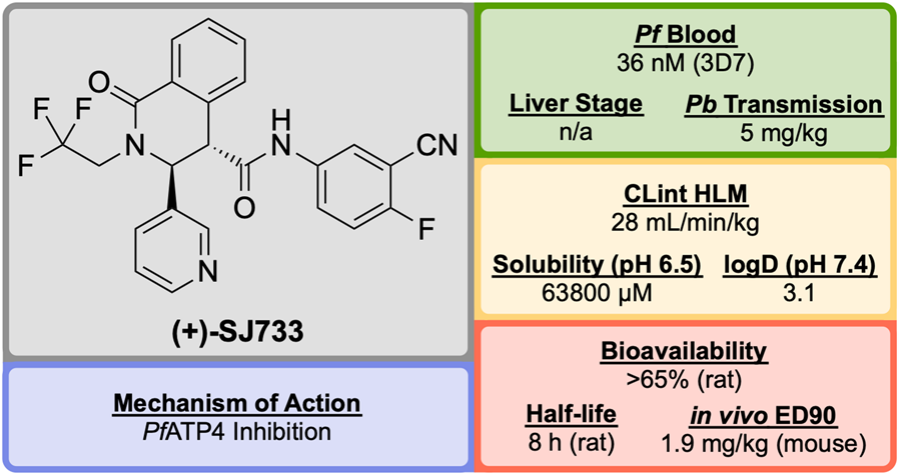
Key biological and physical properties of **(+)-SJ733**. Sexual stage potency and logD kindly provided by K. Guy, personal communication, 2018

Discovered by a partnership between St Jude Children’s Research Hospital and Rutgers University in 2010, **(+)-SJ733** (Fig. 20) is a novel tetrahydroisoquinolone carboxanilide that possesses excellent anti-malarial activity in vivo [99].

**Fig. 21:**
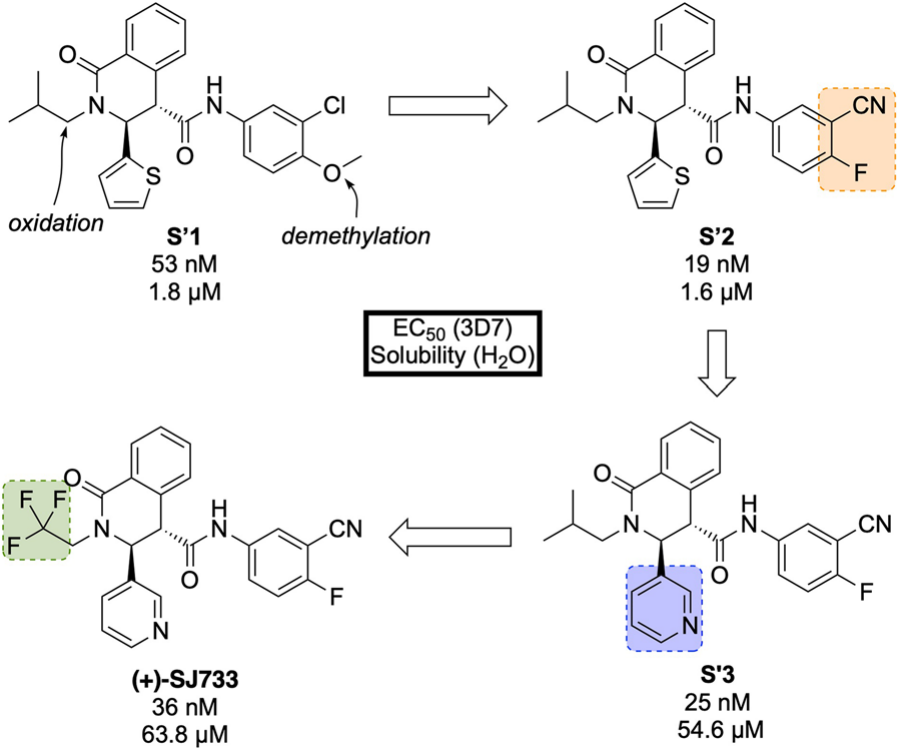
Key stages in the hit to lead pathway of **(+)-SJ733**. Poor metabolic stability of the hit compound was addressed by replacement of the chloro and methoxy groups with cyano and fluoro groups respectively. Further in vivo stability and solubility improvements were made by changing the thiophene to a pyridine. Finally, the *gem*-dimethyl group was substituted with a trifluoromethyl group to eliminate possible metabolic oxidation

High-throughput phenotypic screening of > 300,000 compounds against the 3D7 strain of *P. falciparum* discovered a number of bioactive scaffold types, in which the hit compound (**S′1**, Fig. 21), belonging to the tetrahydroisoquinolone carboxanilide class, was found to have potent in vitro activity (EC$_{50}$ = 53 nM). Metabolic studies using mouse liver microsomes identified the susceptibility of the methoxy group to demethylation. In order to overcome this issue, the aniline substituents were replaced with fluoro and cyano groups (**S′2**), which had an added effect of improving the potency more than twofold. The thiophene group was suspected to be a metabolic hot spot, which was addressed by its replacement with a pyridyl group (**S′3**) resulting also in improved solubility, while maintaining potency. The final metabolically labile group (isobutyl) was replaced with a trifluoromethyl moiety, further improving solubility and maintaining potency to give the lead compound **(+)-SJ733**. The (+)-(3*S*,4*S*) isomer was found to be significantly more potent (EC$_{50}$ = 36 nM) than its (–)-(3*R*,4*R*) enantiomer (EC$_{50}$ = 587 nM) [100].

In the *P. berghei* mouse model, **(+)-SJ733** was found able to cure malaria at doses of 4 $\times$ 100 mg/kg and has an ED$_{90}$ of 1.9 mg/kg. It has shown transmission blocking activity in infected mice with an ED$_{50}$ of 5 mg/kg. It possesses a good safety profile with no cytotoxicity and was found to be more potent in vivo when compared to existing anti-malarials such as artesunate, chloroquine, and pyrimethamine [101].

The molecular target of **(+)-SJ733** has been identified as the P-type Na$^+$–ATPase transporter (*Pf*ATP4, *vide infra*), which has been implicated as a target for a number of other structurally diverse compounds [102]. The compound is currently in the recruiting stage of first-in-human study trials (NCT02661373).

**Fig. 22:**
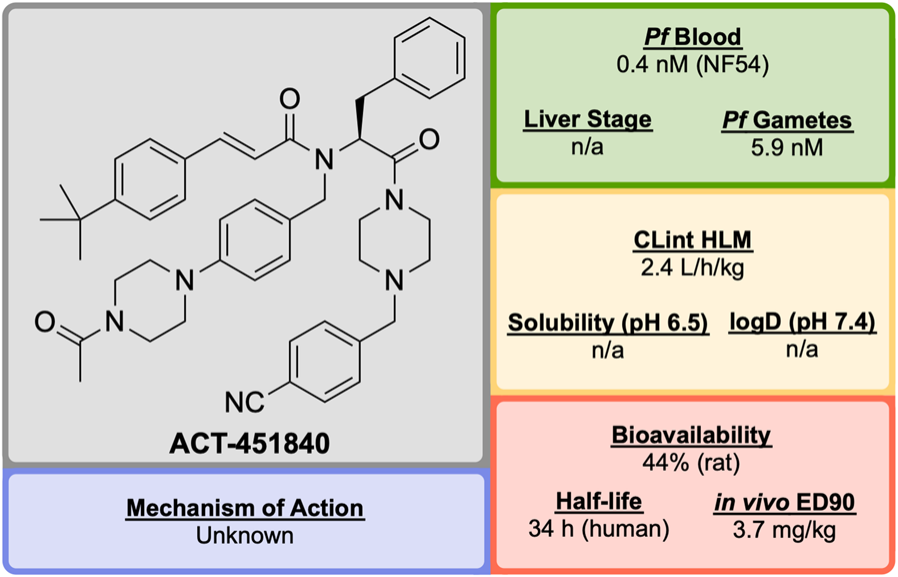
Key biological and physical properties of **ACT-451840**

**ACT-451840** (Fig. 22) is a phenylalanine-based compound that was developed in 2016 through a collaboration between Actelion Pharmaceuticals and the Swiss Tropical and Public Health Institute (STPHI). It has the potential to be a fast-acting drug with a long half-life and has shown efficacy against multiple stages of the *P. falciparum* parasite [103].

**Fig. 23:**
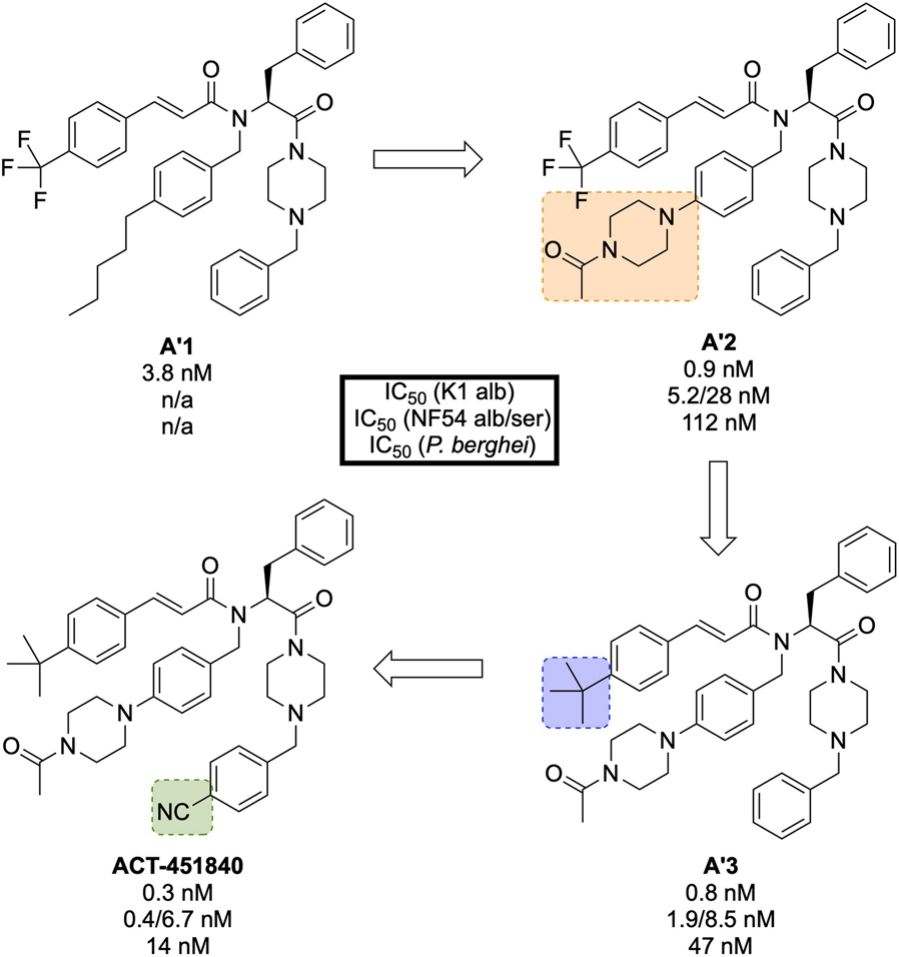
Key stages in the hit to lead pathway of **ACT-451840**. Initial change of the *n*-pentyl group to an acylpiperazine (**A′2**) helped to improve the physicochemical properties. Subsequent introduction of a *tert*-butyl in place of the trifluoromethyl (**A′3**) and a cyano group on the southern phenyl ring resulted in the optimized compound

Initial erythrocyte-based phenotypic screening of $\sim$ 5000 compounds identified **A′1** (Fig. 23) as a highly potent compound against the chloroquine-resistant K1 strain of *P. falciparum* (IC$_{50}$ = 3.8 nM). The SAR studies in this project were unique in that the anti-malarial activity was measured in parallel in two different media: 10% bovine serum albumin and 50% human serum, with the latter used to help identify any potential problems with protein binding at an early stage of the optimization. The stereogenic centre of the amino acid residue proved to be important for the activity, with the (*S*)-isomer showing more than tenfold higher activity compared to the non-natural (*R*)-isomer. Modification of the *n*-pentyl chain to an acylpiperazine group (**A′2**) resulted in an improvement in the physical and chemical properties of the compound. Replacement of the trifluoromethyl group with a *tert*-butyl group (**A′3**) led to improved anti-malarial activity, especially in the presence of human serum proteins. Final installation of a 4-cyano moiety on the southern phenyl ring gave the highly potent (IC$_{50}$ = 0.4 nM) lead candidate **ACT-451840**.

Interestingly, all compounds were found to be significantly less active against *P. berghei* than the human parasite. This is notable since most new anti-malarial drugs in development have shown similar potency against both rodent and human parasites. As a result, for in vivo studies it was crucial to use a humanized *P. falciparum* severe combined immunodeficiency (SCID) mouse model. In the *P. berghei* mouse model, **ACT-451840** showed the ability to cure malaria at doses of 3 $\times$ 300 mg/kg with an ED$_{90}$ of 13 mg/kg. In the *P. falciparum* SCID mouse model it had an ED$_{90}$ of 3.7 mg/kg. The importance of the delivery system for **ACT-451840** was shown through in vivo experiments: a 60 mg dose in corn oil was as effective as a 100 mg dose in a mixture of Tween-EtOH/water = 10:90.

**ACT-451840** has shown activity against multiple parasite life cycle stages of both *P. falciparum* and *P. vivax* [104]. The MoA is suspected to be novel but is currently unknown. **ACT-451840** last completed first-in-human studies in 2014 (NCT02223871) [105] and is currently awaiting a decision to proceed.

### Product development (patient exploratory)

**Fig. 24:**
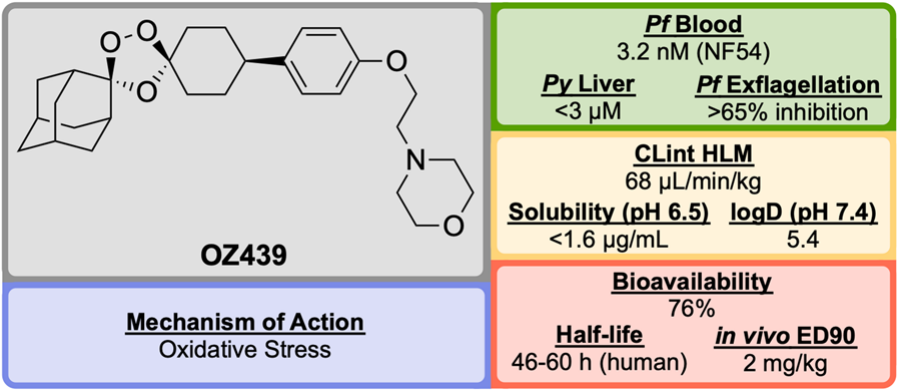
Key biological and physical properties of **OZ439**. In vivo ED_90_ kindly provided by S. Wittlin, personal communication, 2018

Discovered in 2011 by a partnership between Monash University, the University of Nebraska and the STPHI, **OZ439** (Fig. 24) is a synthetic trioxolane that possesses fast-acting curative and transmission-blocking ability, and is active against artemisinin-resistant parasites [106].

**Fig. 25:**
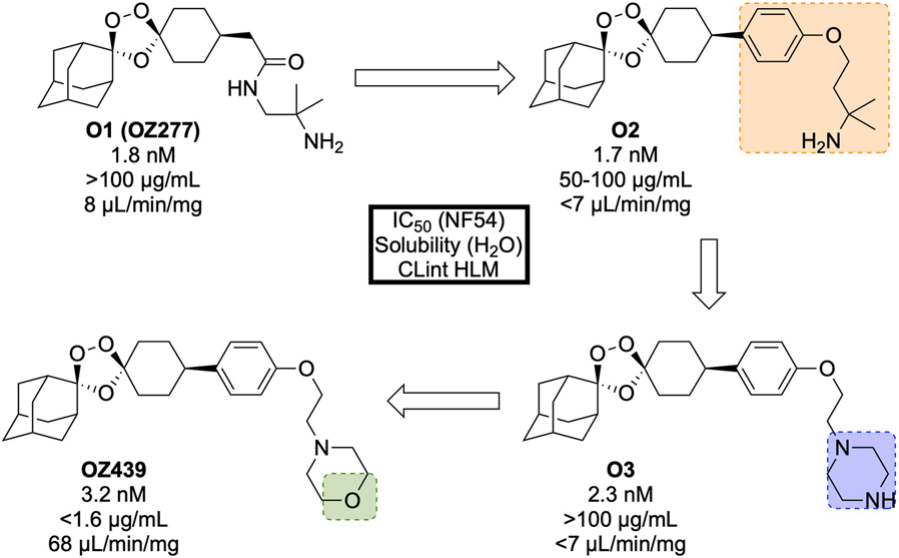
Key stages in the hit to lead pathway of **OZ439**. Initial replacement of the amide linker with a phenyl ether linker resulted in improved exposure while maintaining potency (**O2**). The exposure was further improved by changing the alkylamine chain to a piperazine ring (**O3**). Final replacement of the piperazine ring with a morpholine unit led to the optimized compound **OZ439**, which possessed better curative efficacy in vivo

The discovery of **OZ439** (a.k.a. Artefenomel) stems from the lead optimization of the pre-existing trioxolane **OZ277** (**O1**, Fig. 25, a.k.a. Arterolane), which was discovered in 2004 [107, 108]. As previous studies have shown that the adamantane-peroxide moiety is essential for anti-malarial activity, studies were focused on the eastern portion of the molecule [109–111]. Notably, analogues with variation in this area were found to have largely similar in vitro activities. In order to improve upon the original lead compound **OZ277** (mean survival 11 days, 0 from 5 mice cured), replacement of the amide linker with a phenyl ether linker (**O2**) led to improved exposure and single-dose curative efficacy (mean survival > 30 days, 5 from 5 mice cured when applied in single dose of 30 mg/kg 1 day after infection). An attempt to further improve the exposure saw replacement of the alkylamine chain with a terminal piperazine unit (**O3**), however this led to a significant reduction in curative efficacy (mean survival 17 days, 0 from 5 mice cured). Encouragingly, using morpholine (**OZ439**) resulted in a mean survival of > 30 days, with 5 from 5 mice cured [112]. While **OZ439** has shown excellent curative efficacy, no clear correlations were found between in vitro and in vivo activity or physiochemical properties and exposure. As a result, this new lead compound possess significantly lower solubility and slightly lower potency than **OZ277**.

Unlike other peroxide-containing anti-malarials (e.g. artemisinin), **OZ439** was found to be curative at a single dose of 20 mg/kg in the *P. berghei* mouse model and possessed prophylactic activity superior to mefloquine when a 30 mg/kg dose was applied 24 h prior to malaria infection. **OZ439** also showed full anti-malarial protection when administered 96 h before infection at a dose of 100 mg/kg. Only minor signs of toxicity were observed in a rat model when five consecutive doses of 300 mg/kg were administered with 3 days interval. Conversely, **O2** resulted in the death of 9 from 12 animals when used at the same dose [112]. **OZ439** has shown a significantly longer half-life in humans (about 46–62 h) when compared to the hit compound **OZ277** (3 h) [113–115].

Much like the current peroxide-containing anti-malarials, the precise MoA for **OZ439** has yet to be discovered but it is believed that oxidative stress plays a major role [116, 117]. First-in-human results for **OZ439** were published in 2013 [118] and since then the molecule has progressed into Phase IIb clinical trials with planned completion in 2019 (NCT02497612) [119].

**Fig. 26:**
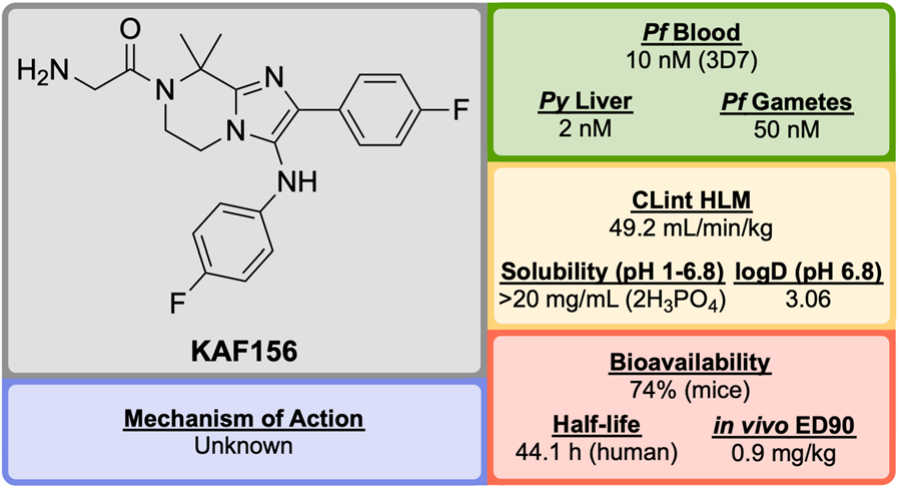
Key biological and physical properties of **KAF156**. Solubility and logD kindly provided by T. Diagana, personal communication, 2018

**KAF156** (Fig. 26) was identified in 2008 by Novartis and The Scripps Research Institute and is part of the second-generation of imidazolopiperazine anti-malarials that potentially possesses a novel MoA and performs as a rapid-acting drug [120].

**Fig. 27:**
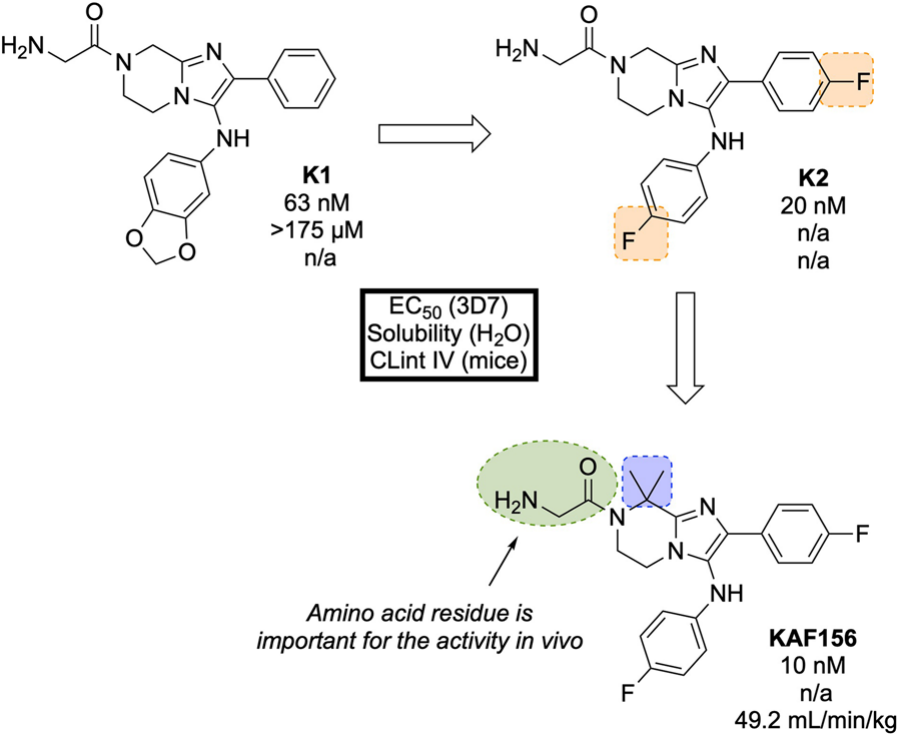
Key stages in the hit to lead pathway of **KAF156**. Potential metabolic stability issues were addressed through fluorine bioisosteres giving **K2**. Introduction of a dimethyl group on the piperazine ring resulted in increased potency and led to the optimized compound

A high-throughput screen of 1.7 million compounds in a *P. falciparum* proliferation assay led to the identification of hit compound **K1** (Fig. 27). In order to protect the metabolically vulnerable positions in the lead compound and to optimize the potency, the benzodioxole and phenyl fragments were replaced with 4-fluorobenzene groups (**K2**). Installation of a dimethyl group in the piperazine ring led to the twofold more potent compound **KAF156** [121, 122]. Notably, removal of the glycine residue from the piperazine ring was shown to increase the in vitro potency further (EC$_{50}$ = 5 nM), but this was accompanied by a lower parasitaemia reduction, indicating the importance of the amino acid side chain for in vivo efficacy [121].

In the causal prophylactic rodent malaria model, a single oral dose of 10 mg/kg administered 2 h before intravenous infection with *P. berghei* sporozoites was shown to be fully protective. **KAF156** has also shown transmission blocking ability in the *P. berghei* model.

The MoA for **KAF156** remains unknown. Through the culturing of resistant strains, mutations have been identified in three genes: *P. falciparum* Cyclic Amine Resistance Locus (*Pf*CARL), UDP-galactose and Acetyl-CoA transporters [58, 123]. **KAF156** is currently in Phase IIb clinical trials, administered in combination with Lumefantrine (NCT03167242).

**Fig. 28:**
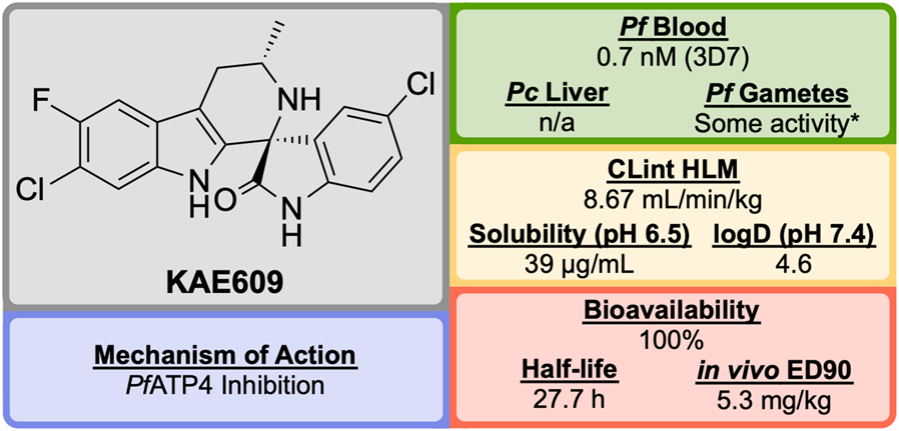
Key biological and physical properties of **KAE609**. *Significant inhibition at 50 and 500 nM doses

**KAE609/Cipargamin/NITD609** (Fig. 28) was discovered by a partnership between Novartis, the STPHI and the Wellcome Trust [124, 125].

**Fig. 29:**
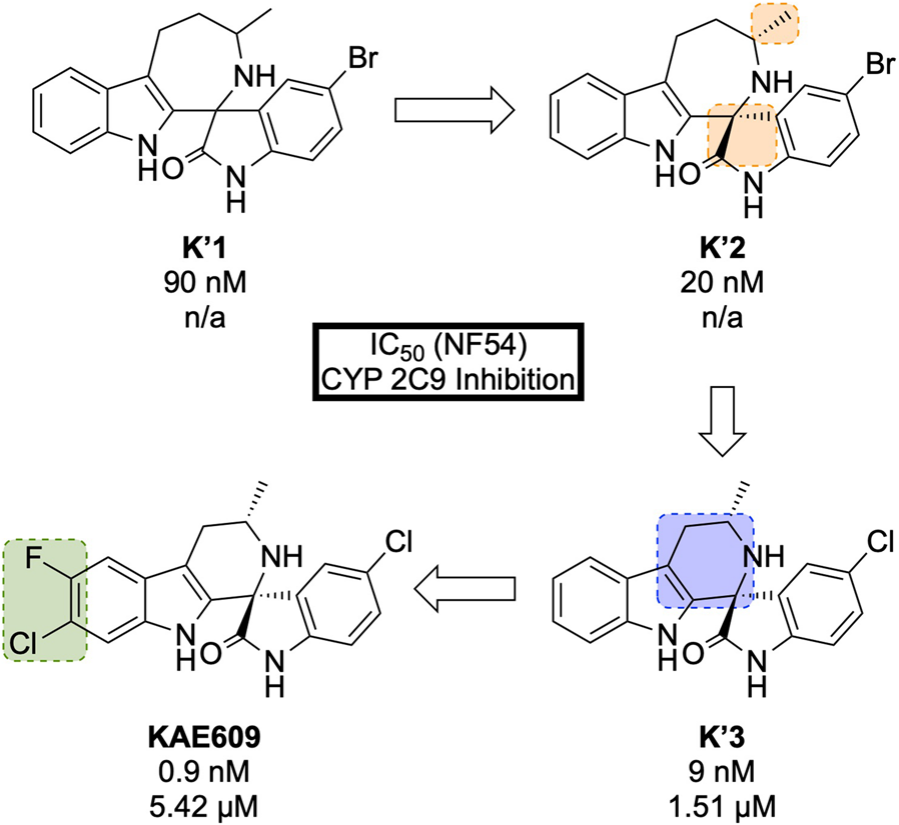
Key stages in the hit to lead pathway of **KAE609**. Resolution of the initial racemic hit (**K′1**) gave the significantly more potent stereoisomer (**K′2**). Reducing the ring size further increased the potency and final halogenation of the indole ring led to the optimized compound **KAE609**

A high-throughput *P. falciparum* proliferation assay identified a racemic spiroazepineindole compound (**K′1**, Fig. 29) which possessed moderate potency against K1 and NF54 strains, as well as potent in vivo efficacy. Confirmation of the hit result by resynthesis of **K′1** resulted in the isolation of a $\sim$ 9:1 diastereomeric ratio favouring the (1*R*,3*S*) and (1*S*,3*R*) pair of enantiomers. Chiral separation of this major pair identified the (1*R*,3*S*) enantiomer **K′2** as being > 250-fold more potent than the (1*S*,3*R*) enantiomer. Reduction of the ring size (**K′3**) facilitated a diastereoselective Pictet–Spengler reaction, which ultimately led to the production of highly potent spiroindolone **KAE609**.

**KAE609** is equipotent against drug-resistant strains and was found to be as effective as artesunate against *P. falciparum* and *P. vivax* isolates [126]. It shows a good safety profile, with low cytotoxicity, cardiotoxicity and mutagenic activity, and is able to clear parasitaemia rapidly in adults with uncomplicated *P. falciparum* or *P. vivax* malaria at a dose of 30 mg/day for 3 days. **KAE609** displays low clearance from the body, has a long half-life and excellent bioavailability.

**KAE609** is one of the key novel compounds that has been found to act through inhibition of *Pf*ATP4 (*vide infra*). Along with a number of other structurally distinct compounds that are also thought to inhibit *Pf*ATP4 as their MoA, **KAE609** has shown great promise as an new anti-malarial candidate and is currently in Phase IIb trials (NCT03334747).

**Fig. 30:**
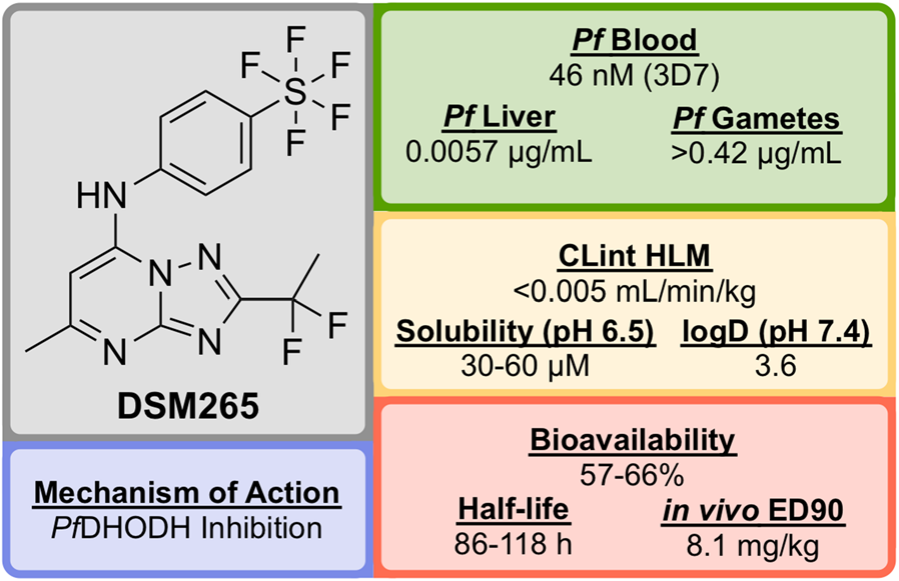
Key biological and physical properties of **DSM265**

**DSM265** (Fig. 30) was discovered through a collaboration between the University of Texas Southwestern, the University of Washington and Monash University [127].

**Fig. 31:**
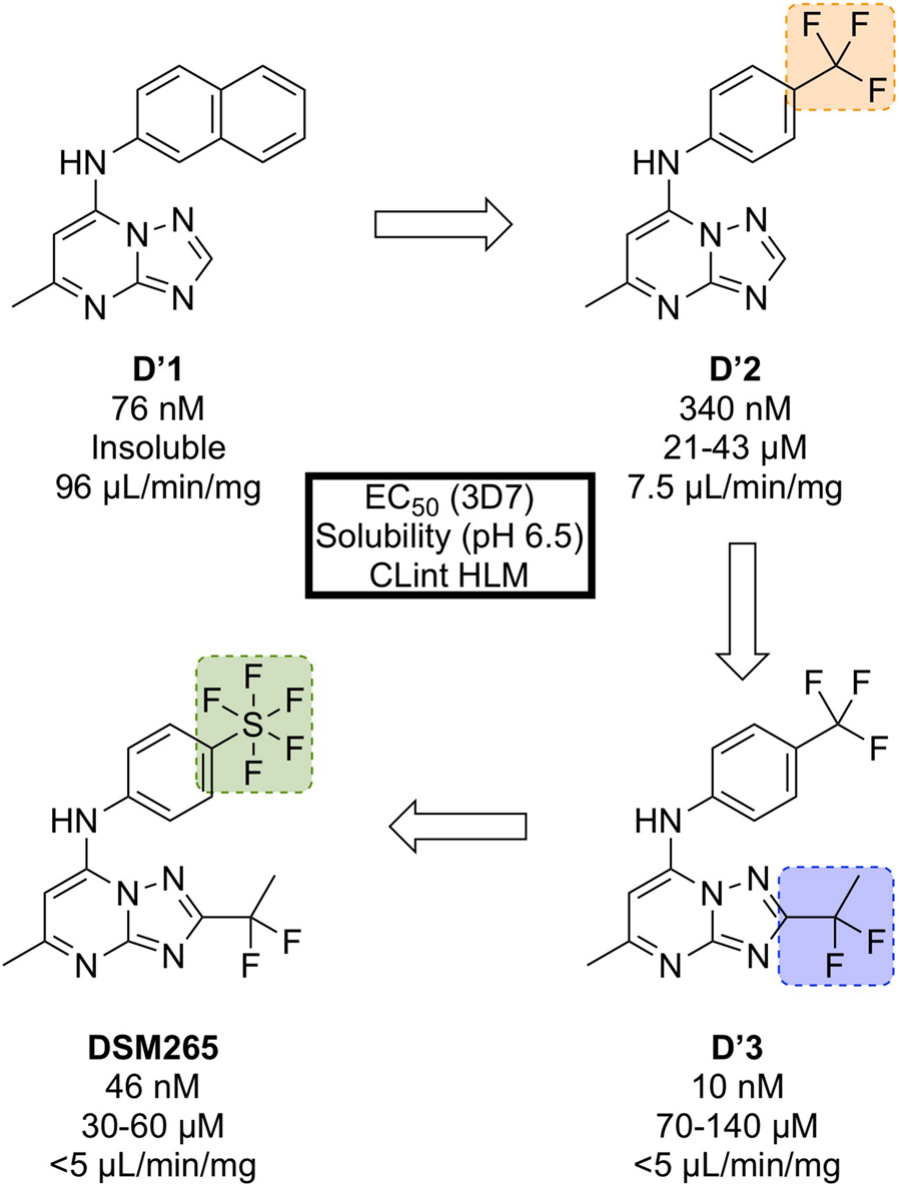
Key stages in the hit to lead pathway of **DSM265**. Replacement of the second phenyl ring in the naphthyl group with a trifluoromethyl group improved solubility. Addition of a 1,1-difluoroethyl group significantly increased the potency and final replacement of the trifluoromethyl group in **D′2** with a pentafluorosulfur moiety led to the optimized compound **DSM265**

Identified in a high-throughput screen of *P. falciparum* dihydroorotate dehydrogenase (*Pf*DHODH)-based enzyme activity, the selective inhibitor **D′1** (Fig. 31) was found. The compound showed high potency but was rapidly metabolized and was inactive in vivo. Substitution of the naphthyl ring with a trifluoromethylphenyl group led to a metabolically stable analogue (**D′2**). Examination of the bound crystal structures of **D′1** and **D′2** with *Pf*DHODH identified key residue interactions which eventually led to the installation of a difluoroethyl group (**D′3**) which significantly improved the potency and solubility. Final modification of the trifluoromethyl group with a pentafluorosulfur group led to the optimized compound **DSM265**.

**DSM265** has been shown to be a highly selective inhibitor of malarial DHODH and is potent against both blood and liver stages of *P. falciparum* and also drug-resistant parasite isolates. It has an excellent safety profile (provides therapeutic concentrations after 8 days of a single oral dose, tolerated in repeat-doses, non-mutagenic, inactive against human enzymes/receptors) and has a very low clearance rate and a long half-life in humans [128]. **DSM265** has completed Phase IIa trials in Peru in patients with *P. falciparum* or *P. vivax* (NCT02123290) and also completed a controlled human malaria infection study in combination with OZ439 (NCT02389348).

**Fig. 32:**
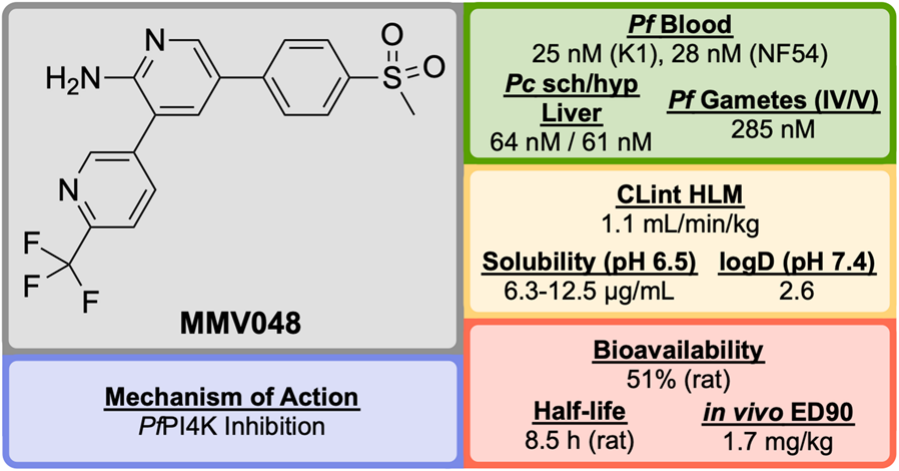
Key biological and physical properties of **MMV048**

Identified in 2012 by a team at the University of Cape Town, South Africa in the same campaign as UCT943, **MMV048** (Fig. 32) is another compound in the 3,5-diaryl-2-aminopyridine class that possesses good prophylactic activity against *Plasmodium cynomolgi* in vivo and has the potential to act as a transmission-blocking drug [84].

**Fig. 33:**
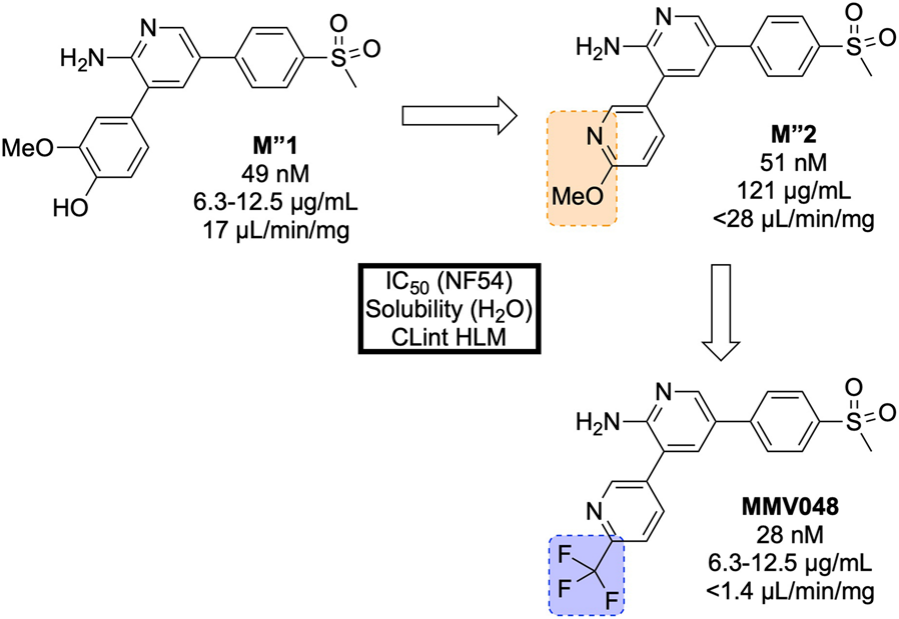
Key stages in the hit to lead pathway of **MMV048**. Initial replacement of the 3-methoxy-4-hydroxyphenyl moiety helped to improve in vivo stability and solubility. Further replacement of the methoxy group with a trifluoromethyl group improved potency and metabolic stability leading to the optimized compound

The initial hit (**M″1**, Fig. 33) showed potent in vitro activity against the NF54 (drug-sensitive) strain of *P. falciparum* (IC$_{50}$ = 49 nM) but suffered from poor solubility and high metabolic clearance. The metabolically labile 3-methoxy-4-hydroxyphenyl moiety was replaced by a methoxypyridyl ring giving the more stable and equipotent compound **M″2**. Further SAR studies revealed that replacement of the methoxy group by a trifluoromethyl group led to the improved potency and stability of **MMV048**.

**MMV048** has shown 99.3% reduction in parasitaemia in the *P. berghei* mouse model at a single dose of 30 mg/kg with no signs of parasites after 30 days (ED$_{90}$ = 1.7 mg/kg). This highlights the potential of **MMV048** to act as a single dose treatment.

The target of **MMV048** is *Pf*PI4K, which was recently revealed as a new MoA for anti-malarial drugs (*vide infra*) [87]. **MMV048** is currently in Phase IIa clinical trials in Ethiopia [58].

## Modes of action

The continual emergence of drug resistant *Plasmodium* strains means that developing new drugs with novel MoAs is important [129]. Below, are summarized some newer MoAs that are currently being pursued and which are exemplified by the compounds described above.

### Translational elongation factor 2 (*Pf*eEF2) inhibitor

The *P. falciparum* translational elongation factor 2 (*Pf*eEF2) is one of the essential factors for eukaryotic protein synthesis, catalysing the translocation of tRNA and mRNA [130]. The overall efficacy of drugs that target this elongation factor may be increased due to *Pf*eEF2 being expressed in multiple stages of the parasite life-cycle [78].

**M5717** has been shown to inhibit *Pf*eEF2 as its MoA, but a binding pocket for **M5717** could not be elucidated from the modelling studies [78]. This MoA was found by culturing blood stage parasites with five times the EC$_{50}$ of **M5717**, leading to resistance in the 3D7, 7G8 and Dd2 strains. Through whole-genome sequencing of these resistance lines, a common mutation was found which identified *Pf*eEF2 as the target.

### P-type Na$^+$–ATPase inhibitor (*Pf*ATP4) inhibitor

The malaria parasite must maintain a low intracellular Na$^+$ concentration to survive, especially in the presence of the high concentration of extracellular sodium ions. In this case, the parasite’s influx of Na$^+$ is regulated by using a P-type ATPase transporter (*Pf*ATP4) that shuttles sodium ions out of the cell. Inhibition of this transporter results in a build up of Na$^+$ inside the parasite, ultimately leading to cell death [102].

A number of structurally diverse compounds (including **(+)-SJ733** and **KAE609**) are thought to target *Pf*ATP4 as their MoA again suggested by the development of resistant mutants and sequencing [124]. The Kirk group have developed a fluorescence assay for this MoA [131], which has been used by the OSM project to implicate *Pf*ATP4 as a MoA for the Series 4 triazolopyrazines [132]. Of note, however, is that this MoA is suggested for a very broad range of chemotypes [133, 134], and there is no direct evidence for binding of these compounds to *Pf*ATP4, the structure of which remains unsolved.

### V-type H$^+$–ATPase inhibitor

At the same time that the parasite is regulating its Na$^+$ concentration through the above mechanism, it also imports H$^+$ through the same pathway. To regulate this increasing H$^+$ concentration and maintain an intracellular pH of $\sim$ 7.3, the parasite uses a complementary V-type ATPase transporter to efflux H$^+$ [135].

**MMV253** has been shown to inhibit the V-type H$^+$ ATPase as its MoA through mutant selection and whole-genome sequencing [81].

### Phosphatidylinositol 4-kinase (*Pf*PI4K) inhibitor

Phosphatidylinositol 4-kinase (PI4K) is a eukaryotic enzyme that phosphorylates lipids, allowing them to regulate intracellular signalling and trafficking [87]. Inhibition of the ATP-binding pocket of PI4K leads to disruption of the intracellular distribution of PI4-phosphate (PI4P), which in turn results in decreased late-stage parasite development.

By culturing resistant lines and identifying the genomic mutations, two compounds (**UCT943** and **MMV048**) have been found to inhibit the *Pf*PI4K enzyme [87].

### Dihydroorotate dehydrogenase (*Pf*DHODH) inhibitor

To be able to replicate in the human erythrocyte, the *Plasmodium* parasite requires pyrimidines, which are essential metabolites in all cells. As the parasite is unable to use endogenous pyrimidines, it must synthesize them de novo. A key step in the biosynthesis of pyrimidines is the oxidation of dihydroorotate to produce orotate, a reaction that is catalysed by the enzyme dihydroorotate dehydrogenase (DHODH). By inhibiting this enzyme, the malaria parasite can no longer obtain the required metabolites to survive, and is killed [136].

The *P. falciparum* D10 strain (chloroquine sensitive) has been known to be less susceptible to bc_1_ complex inhibitors and *Pf*DHODH inhibitors. Addition of proguanil to this strain is known to restore the sensitivity to bc_1_ complex inhibitors but not to *Pf*DHODH inhibitors. This cell line was found to be resistant to **DSM265**, regardless of whether proguanil was present or not, which identifies inhibition of *Pf*DHODH as its MoA [127, 137].

### Dihydrofolate reductase (*Pf*DHFR) inhibitor

Similar to the above case, the malaria parasite also requires folates in order to maintain their high rate of replication. The parasite is able to scavenge folates or synthesize them *de novo*. Dihydrofolate reductase (DHFR) is an enzyme that catalyses a step required for the recycling of folates, which in turn are used in the synthesis of thymidylate, purines and methionine [138].

*Pf*DHFR is a known target of a number of well known antifolate anti-malarial drugs including cycloguanil and pyrimethamine. Through examinations of the key interactions in the cocrystal structure of known inhibitors with the enzyme, **P218** was designed as a potent inhibitor [98].

## Conclusions

Although the rate of malaria related deaths has declined over the past few years, the progress is beginning to slow. With the recent emergence of resistance to current front-line artemisinin-based combination therapy, the need for the discovery of new anti-malarials that can act through novel mechanisms of action has been pushed firmly to the top of the development agenda.

Over the past few years, high-throughput screens have identified a number of novel chemotypes that have since been developed into highly promising anti-malarial candidates. Crucially, many of these compounds have demonstrated novel MoAs which are essential if future drugs are to succeed. This is wonderful progress, and testament to the hard work of the groups involved.

The discovery of these novel MoAs will pave the way for the development of future anti-malarials. The exploration of further novel MoAs has become a possibility through the use of in vitro evolution and whole-genome analysis (IVIEWGA), which uses genome-base target discovery methods on compounds identified from the more common phenotypic screens [139]. In all such future development, creativity and ingenuity in the hit to lead campaigns will continue to be needed if people are to remain able to combat this formidable disease.

The pace of research progress is high, meaning updates to reviews such as this will always be needed (Additional file 3). It is proposed that derivatives of this open access review, combined with data in relevant databases such as the one currently in development by the Guide to Pharmacology [140], could aim to serve as more “living” sources of information for compounds in development and their potential targets.

## Additional files

**Additional file 1.** Compounds reference list. List of SciFinder reference dois for each compound described in the Future section of this review.

**Additional file 2.** Biological data for compounds. Tabulation of biological potencies and pharmacokinetic properties for each compound described in Future section of this review.

**Additional file 3.** SMILES strings and CID numbers for compounds. List of SMILES strings and CID numbers for every compound described in this review.

## Abbreviations

ACT: artemisinin-based combination therapy
CG: cycloguanil
CL_int_: intrinsic clearance
CQ: chloroquine
DDU: Drug Discovery Unit
EC_50_: half maximal effective concentration
ED_90_: 90% effective dose
GSK: GlaxoSmithKline
hERG: human ether-à-go-go-related gene
HLM: human liver microsome
IC_50_: half maximal inhibitory concentration
i.p.: intraperitoneally
IVIEWGA: in vitro evolution and whole-genome analysis
IVIVC: in vitro-in vivo correlation
MLEM: Model List of Essential Medicines
MLM: mouse liver microsome
MMV: Medicines for Malaria Venture
MoA: mode/mechanism of action
mRNA: messenger ribonucleic acid
MTX: methotrexate
OSM: Open Source Malaria
*P.*: *Plasmodium*
*p.o.*: (per os) orally
*Pb*: *Plasmodium berghei*
*Pc*: *Plasmodium* cynomolgi
*Pf*: *Plasmodium falciparum*
*Pf *ATP4: *Plasmodium falciparum* ATPase transporter
*Pf *CARL: *Plasmodium falciparum* cyclic amine resistance locus
*Pf *CPSF3: *Plasmodium falciparum* cleavage and polyadenylation specificity factor
*Pf *DHFR: *Plasmodium falciparum* dihydrofolate reductase
*Pf *DHODH: *Plasmodium falciparum* dihydroorotate dehydrogenase
*Pf *eEF2: *Plasmodium falciparum* translational elongation factor 2
*Pf *PI3K: *Plasmodium falciparum* phosphatidylinositol-3-kinase
*Pf *PI4K: *Plasmodium falciparum* phosphatidylinositol-4-OH kinase
*Py*: *Plasmodium yoelli*
PYR: pyrimethamine
*q.d.*: (*quaque die*) one a day
RCQ(s): reversed chloroquine(s)
SAR: structure activity relationship
SCID: severe combined immunodeficiency
STPHI: Swiss Tropical and Public Health Institute
TAP: triaminopyrimidine
TQ: tafenoquine
tRNA: transfer ribonucleic acid
WHO: World Health Organization
